# From Oxyntomodulin to Retatrutide: A Translational Review of Metabolic Polyagonism

**DOI:** 10.3390/ijms27219654

**Published:** 2026-10-28

**Authors:** Héctor Iván Saldívar-Cerón, Daniela Maldonado-Trejo, Quetzal Chavez-Castañeda, Ana Lorena Mercado-Guzmán, Angela de Jesús León-Barrita, Isela Alejandra López-García, Alejandra Hernández-Solís, Andrés Moranchel Ramírez-Mendoza, Daniela Alejandra Terrazas-Delgado, Ari Evelyn Castañeda-Ramírez, Efrén Quiñones-Lara, Brenda Ofelia Jay-Jímenez, Glustein Pozo-Molina, Sonia León-Cabrera

**Affiliations:** 1 Unidad de Remisión de Diabetes Mellitus (URDM), Carrera de Médico Cirujano, Facultad de Estudios Superiores-Iztacala, Universidad Nacional Autónoma de México, Tlalnepantla 54090, Estado de Mexico, Mexico; danmt1303@gmail.com (D.M.-T.); chavezcastanedaquetzal@gmail.com (Q.C.-C.); loremerguz01@gmail.com (A.L.M.-G.); angybarritaleon1303@gmail.com (A.d.J.L.-B.); iselalejandra14med@gmail.com (I.A.L.-G.); hernandez.solis.alejandra.501@gmail.com (A.H.-S.); moranchel004@gmail.com (A.M.R.-M.); danielaterrazas0309@gmail.com (D.A.T.-D.); aricasra206@gmail.com (A.E.C.-R.); 2 Departamento de Biomedicina Molecular, Centro de Investigación y de Estudios Avanzados del Instituto Politécnico Nacional (CINVESTAV-IPN), Ciudad de Mexico 07360, Mexico; efren.quinones@cinvestav.mx; 3 Centro Internacional de Simulación y Entrenamiento en Soporte Vital Iztacala (CISESVI), Facultad de Estudios Superiores-Iztacala, Universidad Nacional Autónoma de México, Tlalnepantla 54090, Estado de Mexico, Mexico; mcbrendajay@iztacala.unam.mx; 4 Laboratorio de Genética y Oncología Molecular, Carrera de Médico Cirujano, Facultad de Estudios Superiores-Iztacala, Universidad Nacional Autónoma de México, Tlalnepantla 54090, Estado de Mexico, Mexico; glustein@iztacala.unam.mx; 5 Unidad de Biomedicina (UBIMED), Carrera de Médico Cirujano, Facultad de Estudios Superiores-Iztacala, Universidad Nacional Autónoma de México, Tlalnepantla 54090, Estado de Mexico, Mexico; soleonca@comunidad.unam.mx

**Keywords:** oxyntomodulin, retatrutide, glucagon receptor, GIP receptor, GLP-1 receptor, Class B1 GPCR, polyagonism, receptor pharmacology, mixed-meal tolerance test, treatment-response heterogeneity

## Abstract

Oxyntomodulin (OXM), an endogenous proglucagon-derived peptide with dual glucagon-like peptide-1 receptor (GLP-1R) and glucagon receptor (GCGR) agonism, provides a physiological precedent for contemporary multi-receptor pharmacology. Recognition that GCGR signaling regulates not only hepatic glucose production but also amino acid turnover, lipid metabolism, substrate oxidation, and energy expenditure prompted the development of engineered GLP-1R/GCGR co-agonists, including cotadutide, mazdutide, survodutide, and pemvidutide. Retatrutide extends this strategy by integrating GIPR, GLP-1R, and GCGR agonism within a single long-acting peptide and has produced substantial improvements in body weight, glycemic control, and cardiometabolic parameters in phase 2 and phase 3 trials. This review examines the evolutionary and structural relationships of the proglucagon–Class B1 GPCR system, OXM biosynthesis and receptor pharmacology, the mechanistic rationale for therapeutic GCGR agonism, and the molecular and clinical development of dual and triple agonists. We further address receptor balance, the GIPR agonism–antagonism paradox, receptor-specific causal attribution, and the unresolved limitations of translating molecular pharmacology into durable clinical benefit. Finally, we propose an Activity–Exposure–Context framework for interpreting polyagonist pharmacology and discuss prospective enteroinsular phenotyping using standardized mixed-meal testing as a testable strategy to investigate interindividual variability in therapeutic response.

## 1. Introduction

Obesity and type 2 diabetes mellitus (T2DM) involve persistent disturbances in energy balance, glucose regulation, and nutrient partitioning, with clinical consequences extending beyond body weight and fasting glucose to cardiovascular and metabolic liver disease [1,2,3]. The biological response to weight reduction further complicates long-term treatment: hunger can remain elevated after weight loss [4], while energy requirements decline, including an adaptive component that cannot be explained solely by the loss of metabolically active tissue [5,6]. These compensatory responses make maintenance of reduced weight a distinct therapeutic challenge rather than a simple continuation of initial weight loss.

Long-acting glucagon-like peptide-1 receptor (GLP-1R) agonists established that pharmacological manipulation of nutrient-responsive endocrine pathways can produce substantial weight reduction alongside glycemic improvement [7,8,9]. Semaglutide and the dual glucose-dependent insulinotropic polypeptide receptor (GIPR)/GLP-1R agonist tirzepatide extended this therapeutic paradigm [7,10,11,12]. Their clinical success encouraged the development of unimolecular multi-receptor agonists but did not establish that adding a receptor target necessarily improves efficacy or safety. Clinical comparisons therefore remain specific to the molecules, doses, populations, exposure profiles, and endpoints tested.

Reduced food intake is an important mechanism of established incretin-based obesity therapies [13,14,15]. This has renewed interest in complementary pathways affecting hepatic substrate handling and, potentially, energy expenditure [16,17]. Glucagon receptor (GCGR) agonism is particularly relevant because it introduces both an opportunity and a liability: GCGR signaling can promote hepatic lipid oxidation, amino acid turnover, and substrate mobilization [17,18,19], while stimulation of hepatic glucose production can oppose glycemic control [18,19,20]. The therapeutic question is therefore not whether glucagon is intrinsically beneficial or detrimental, but whether selected metabolic actions of GCGR signaling can be harnessed within a pharmacological context that preserves glycemic control.

Oxyntomodulin (OXM), an endogenous proglucagon-derived peptide, provides a physiological example of dual GLP-1R/GCGR agonism [21,22,23]. Early human studies demonstrated reductions in energy intake and body weight following pharmacological OXM administration [24,25,26], whereas receptor-based experiments subsequently helped distinguish GLP-1R- and GCGR-dependent components of its metabolic actions [23,27,28,29]. Engineered GLP-1R/GCGR co-agonists translated this endogenous pharmacological precedent into long-acting molecules with tunable receptor activity [28,29], and retatrutide extended this strategy by incorporating GIPR agonism within a single peptide [30,31]. This trajectory is conceptual rather than genealogical: contemporary co-agonists exploit pharmacological principles exemplified by OXM but do not necessarily share an OXM-derived chemical scaffold.

This review integrates the evolutionary and structural biology of the proglucagon–Class B1 G protein-coupled receptor system with native OXM pharmacology, the molecular differentiation of GLP-1R/GCGR co-agonists, and the development of GLP-1R/GIPR/GCGR triagonism culminating in retatrutide. Particular emphasis is placed on how receptor affinity, potency, efficacy, signaling, pharmacokinetic exposure, and biological context collectively shape the metabolic phenotype of multi-receptor agonists. We further examine the mechanistic paradox whereby both GIPR agonism and antagonism can enhance weight reduction, distinguish whole-drug clinical efficacy from receptor-specific causal attribution, and propose an Activity–Exposure–Context framework for interpreting polyagonist pharmacology. Finally, we discuss whether standardized mixed-meal testing coupled with enteroinsular phenotyping could provide a testable precision-pharmacology strategy for investigating interindividual heterogeneity in therapeutic response. A dedicated critical appraisal considers molecular design constraints, unmet clinical endpoints, safety and implementation barriers, and the validation still required for the proposed framework.

### Literature Search and Evidence Interpretation

This article was designed as a narrative review. Targeted searches of PubMed/MEDLINE, Scopus, Web of Science, and ClinicalTrials.gov were performed using combinations of terms related to “oxyntomodulin,” “proglucagon,” “glucagon receptor,” “GLP-1 receptor,” “GIP receptor,” “Class B1 GPCR,” “dual agonist,” “co-agonist,” “triagonist,” “retatrutide,” “cotadutide,” “mazdutide,” “survodutide,” “pemvidutide,” “obesity,” “type 2 diabetes,” “MASLD,” and “MASH.” Additional targeted searches addressed GIPR agonism and antagonism, receptor signaling and trafficking, treatment-response heterogeneity, and mixed-meal testing. Priority was given to original mechanistic and structural studies, randomized clinical trials, and relevant high-quality reviews. Because this was a narrative review, no formal risk-of-bias assessment or quantitative evidence synthesis was performed. Peer-reviewed publications, structural database deposits, and sponsor communications are distinguished where relevant. Metabolic dysfunction-associated steatotic liver disease (MASLD) and metabolic dysfunction-associated steatohepatitis (MASH) follow the multisociety nomenclature consensus; historical terminology is retained in cited article titles [32].

During revision, a supplementary targeted PubMed search was performed on 20 October 2026 to improve retrieval across changing liver-disease terminology. The title/abstract search combined individual agent names and development codes (oxyntomodulin, cotadutide/MEDI0382, mazdutide/IBI362, survodutide/BI 456906, pemvidutide/ALT-801, and retatrutide/LY3437943) or the terms “dual agonist” and “triple agonist” with NAFLD, NASH, MAFLD, MASLD, MASH, and their expanded disease names, including nonalcoholic and non-alcoholic spelling variants. No publication-year restriction or date-dependent exclusion of historical terms was applied. NAFLD/NASH were included as historical retrieval terms, MAFLD as a related alternative diagnostic framework, and MASLD/MASH as the current terminology used in this review [32,33]. These terms improve search sensitivity but do not imply identical diagnostic populations: original eligibility criteria and disease labels were retained when interpreting individual studies, without retrospective reclassification solely by publication year. This supplementary search specifically addressed hepatic evidence; it was not a new systematic search of all databases listed above.

A further targeted literature update was performed on 22 October 2026 using the terms “SYNCHRONIZE-2” and “survodutide phase 3” to locate the newly published trial report and check its PubMed and New England Journal of Medicine records. This update added the phase 3 report in adults with T2DM published on 1 October 2026 to Section 5.2. It was a focused supplementary update, not a new systematic search of all databases or a general extension of the evidence-search cutoff.

## 2. Evolutionary Diversification of the Proglucagon–Class B1 GPCR System

The proglucagon system exemplifies the evolutionary diversification of peptide hormones from a common precursor into ligands with distinct but interconnected metabolic functions [34,35,36]. Tissue-specific processing of proglucagon generates glucagon, GLP-1, GLP-2, OXM, glicentin, and the major proglucagon fragment, whereas GIP originates from a distinct precursor [36,37,38,39,40]. In parallel, GCGR, GLP-1R, GLP-2R, and GIPR belong to an evolutionarily related subgroup of Class B1 G protein-coupled receptors that retains substantial conservation in ligand recognition and receptor activation while supporting specialized physiological functions [34,37,38,41]. This combination of ligand diversification and conserved receptor architecture provides the evolutionary and structural framework for understanding both the endogenous cross-reactivity of peptides such as OXM and the rational design of modern dual and triple receptor agonists [31,41,42,43].

### 2.1. Tissue-Specific Processing of Proglucagon

The human GCG gene encodes preproglucagon, which is converted to proglucagon following removal of its N-terminal signal peptide. Proglucagon subsequently undergoes tissue-specific post-translational processing by prohormone convertases, generating distinct but structurally related bioactive peptides [39,40]. In pancreatic α-cells, prohormone convertase 2 (PC2) predominantly processes proglucagon to generate glucagon and the major proglucagon fragment (MPGF) [40]. In contrast, enteroendocrine L cells of the intestine predominantly express prohormone convertase 1/3 (PC1/3), which generates glicentin, OXM, GLP-1, GLP-2, and related peptide products [39,40]. Thus, differential processing of a common precursor enables the proglucagon system to generate tissue-specific endocrine outputs without requiring separate genes for each peptide.

This molecular organization supports complementary physiological responses to fasting and nutrient ingestion. Pancreatic glucagon promotes hepatic glucose production and contributes to amino acid catabolism, ureagenesis, lipid oxidation, and substrate mobilization during fasting and metabolic demand [19,20,44]. By contrast, intestinal GLP-1 couples nutrient ingestion to glucose-dependent insulin secretion, regulation of glucagon secretion, gastrointestinal motility, satiety, and reduced energy intake [8,40]. GLP-2 primarily acts on the gastrointestinal tract, where it promotes intestinal growth, mucosal integrity, nutrient absorption, and adaptive responses of the intestinal epithelium [45,46,47]. OXM occupies a distinctive position within this processing network because a single intestinal proglucagon-derived peptide retains agonist activity at both GLP-1R and GCGR [21,22,23]. The physiological roles of glicentin and MPGF remain less completely defined [48]. Moreover, the metabolic effects of administered OXM should not be equated with proof that endogenous circulating OXM produces equivalent receptor engagement in humans [21,22].

### 2.2. Functional Diversification Across Vertebrates

Evolutionary diversification of the glucagon peptide family generated structurally related peptides with distinct physiological functions across vertebrates [34,35,36]. In mammals, glucagon primarily coordinates metabolic adaptation to fasting through GCGR-dependent regulation of hepatic glucose production, amino acid catabolism, ureagenesis, lipid oxidation, and substrate mobilization [19,20,44]. In mammalian physiology, GLP-1 has prominent postprandial functions linking nutrient ingestion to glucose-dependent insulin secretion, regulation of glucagon secretion, gastrointestinal motility, satiety, and energy intake [8,40], whereas GLP-2 primarily supports intestinal growth, epithelial integrity, nutrient absorption, and intestinal adaptation [45,46,47]. These functional differences illustrate how diversification within a common peptide family can support complementary aspects of fasting, feeding, and gastrointestinal physiology.

Like other proglucagon-derived peptides, OXM is generated through tissue-specific processing of the common precursor; its distinctive pharmacological feature is dual receptor activity [21,35,39]. Its 37-amino acid sequence contains the complete 29-amino acid glucagon sequence followed by an eight-residue C-terminal extension, preserving sufficient structural compatibility to activate both GCGR and GLP-1R [21,22,23]. OXM is less potent than glucagon at GCGR and less potent than GLP-1 at GLP-1R, but its ability to engage both receptors within a single endogenous peptide provides a physiological example of receptor cross-reactivity within the proglucagon system [21,22,23]. This dual pharmacology links anorectic and insulinotropic GLP-1R signaling with GCGR-dependent regulation of hepatic substrate metabolism and energy balance [21,22,23,27].

Comparative endocrinology further indicates that the physiological roles of glucagon-related peptides and their receptors have diversified across vertebrate lineages rather than remaining fixed throughout evolution [34,35,36,49]. Differences in peptide processing, receptor repertoires, ligand selectivity, and metabolic function have been described across species [34,36,49]. Thus, the contemporary mammalian actions of glucagon, GLP-1, GLP-2, and OXM represent specialized outcomes within an evolutionarily conserved peptide–receptor system. The persistence of structural relatedness despite functional diversification is particularly relevant to metabolic pharmacology because it provides the molecular context in which endogenous cross-reactivity—and subsequently engineered multi-receptor agonism—can occur.

### 2.3. Receptor Diversification and Conserved Structural Compatibility

GCGR, GLP-1R, GLP-2R, and GIPR belong to an evolutionarily related subgroup of Class B1 G protein-coupled receptors characterized by a large N-terminal extracellular domain (ECD) coupled to a seven-transmembrane domain (TMD) [34,41,50,51]. Peptide recognition follows the general two-domain architecture characteristic of Class B1 GPCRs: the C-terminal region of the peptide is primarily engaged by the ECD, whereas the peptide N-terminus penetrates the transmembrane core and contributes directly to receptor activation [50,51,52]. Conservation of this binding architecture provides a common structural framework for ligand recognition, while sequence and conformational differences within the ECD, extracellular loops, and transmembrane binding pocket confer receptor-specific pharmacology [42,50,51,52].

This combination of structural conservation and receptor-specific accommodation enables related peptide ligands to display different degrees of cross-reactivity. OXM provides the most relevant endogenous example for metabolic pharmacology, activating both GLP-1R and GCGR despite lower potency than the corresponding cognate ligands [21,22,23]. Glucagon can also activate GLP-1R under selected experimental conditions, further illustrating that ligand recognition within this receptor subgroup is not governed by absolute one-ligand–one-receptor specificity [23,52]. Structural studies of engineered dual agonists have subsequently shown how a single peptide can preserve a shared receptor-binding architecture while establishing distinct interactions with GLP-1R and GCGR [42].

Recent structural deposits provide additional examples of OXM-bound receptor complexes: GLP-1R–Gs (PDB 9MZE, 2.20 Å) and GCGR–Gs (PDB 9N0E, 2.30 Å) [53,54]. The ligand deposited in 9MZE contains Lys34 rather than the Arg34 present in the human OXM sequence; the deposit should therefore not be described as an exact structural determination of that human sequence [53,55]. These database records are distinguished from peer-reviewed structural reports.

This conserved molecular compatibility created an exploitable design space for medicinal chemistry. Sequence substitutions can modify proteolytic stability, receptor affinity, potency, efficacy, and relative activity across GLP-1R, GCGR, and GIPR, while lipidation and other chemical modifications can independently alter pharmacokinetic exposure [28,29,30,31,41,42]. Consequently, modern dual and triple agonists are not defined simply by the number of receptors they activate, but by the combination of receptor-specific pharmacology and sustained exposure achieved by the engineered molecule. Retatrutide exemplifies this principle: a single peptide can engage GIPR, GLP-1R, and GCGR through a combination of conserved Class B1 recognition features and receptor-specific molecular interactions [31,43].

### 2.4. GIP Within the Related Receptor Family

GIP is encoded by a gene distinct from GCG and is secreted predominantly by enteroendocrine K cells of the proximal small intestine in response to nutrient ingestion [37,56]. GIP is a major component of the enteroinsular axis, enhancing glucose-dependent insulin secretion through activation of GIPR and contributing to postprandial nutrient handling [37,56]. Beyond the pancreatic β-cell, GIPR is expressed in multiple metabolically relevant tissues and cell populations, including adipose and neuronal compartments, where its activation can influence lipid handling, energy balance, and feeding behavior [56,57,58,59]. The physiological consequences of GIPR signaling are therefore strongly dependent on cellular context, metabolic state, and duration of receptor activation [56,57,58,59].

Although GIP does not arise from proglucagon processing, GIPR belongs to the same evolutionarily related Class B1 GPCR subgroup as GLP-1R and GCGR and shares key structural principles of peptide recognition and receptor activation [34,41,50,51,52]. This receptor-level relationship is pharmacologically important because a single engineered peptide can accommodate the conserved binding architecture of GIPR, GLP-1R, and GCGR while retaining receptor-specific interactions [31,43]. GIPR can therefore be integrated into unimolecular polyagonists not because GIP is a product of proglucagon processing, but because its receptor occupies a structurally compatible region of Class B1 GPCR pharmacology.

The clinical development of tirzepatide established that sustained GIPR/GLP-1R co-agonism can produce substantial improvements in glycemic control and body weight [11,12]. However, the contribution of GIPR cannot be inferred simply from differences between tirzepatide and selective GLP-1R agonists, because these molecules also differ in sequence, receptor pharmacology, pharmacokinetic exposure, dose, and treatment context. This distinction becomes particularly important for retatrutide, in which GIPR activity is integrated with GLP-1R and GCGR agonism within the same molecular scaffold [31]. The mechanistic role of GIPR in multi-receptor therapy therefore requires consideration of receptor activity, exposure, and tissue context rather than attribution based solely on whole-drug clinical efficacy.

### 2.5. From Evolutionary Relationships to Pharmacological Design

The evolutionary diversification of proglucagon-derived peptides and their receptors generated related ligands with distinct physiological functions while preserving sufficient structural compatibility for cross-receptor recognition [34,35,36,37,38]. OXM exemplifies this principle at the endogenous level by demonstrating that a single proglucagon-derived peptide can engage both GLP-1R and GCGR [21,22,23]. At the receptor level, conservation of the Class B1 binding architecture provides a molecular basis for this cross-reactivity, whereas differences within extracellular and transmembrane interaction surfaces preserve receptor-specific pharmacology [42,50,51,52]. GIP extends this framework beyond the proglucagon-derived peptide family because, although encoded independently, GIPR retains sufficient structural compatibility with GLP-1R and GCGR to be incorporated into the same multi-receptor design space [31,41,43].

Medicinal chemistry transformed this endogenous and structural compatibility into a tunable pharmacological strategy. Amino acid substitutions can alter receptor affinity, potency, efficacy, proteolytic stability, and relative activity across GLP-1R, GCGR, and GIPR, whereas lipidation and other conjugation strategies can prolong systemic exposure and modify the temporal profile of receptor engagement [28,29,30,31,41]. Consequently, modern polyagonism is better understood not as replication of a native hormone, but as the deliberate engineering of shared receptor recognition, receptor-specific activity, and pharmacokinetic exposure within a single molecular scaffold. GLP-1R/GCGR co-agonists represent the first therapeutic implementation of this principle, whereas GLP-1R/GIPR/GCGR triagonists extend it to three related Class B1 receptors [28,29,30,31]. This transition from endogenous receptor cross-reactivity to quantitatively engineered multi-receptor pharmacology provides the conceptual foundation for the development of retatrutide. Figure 1 summarizes precursor processing, the human OXM sequence, and the dual-receptor concept.

## 3. Oxyntomodulin as an Endogenous Precedent for Polyagonism

OXM occupies a distinctive position within the proglucagon system because dual GLP-1R/GCGR agonism is encoded within a single endogenous peptide [21,22,23]. Its pharmacological relevance derives from the convergence of three properties: structural continuity with glucagon, measurable agonist activity at both GLP-1R and GCGR, and metabolic effects on food intake, glucose regulation, body weight, and energy balance following exogenous administration [21,22,23,24,25,27]. OXM therefore provides an endogenous pharmacological precedent for unimolecular receptor co-activation and a mechanistic starting point for understanding the subsequent development of engineered GLP-1R/GCGR co-agonists.

### 3.1. Discovery and Biochemical Structure

Early investigations of intestinal glucagon-like immunoreactivity revealed that the gastrointestinal tract contained glucagon-related peptides that were immunologically and biochemically distinct from pancreatic glucagon [62]. These observations contributed to the historical concept of enteroglucagon, which initially encompassed heterogeneous glucagon-immunoreactive material rather than a single molecular entity [62]. Subsequent isolation and structural characterization by Bataille and colleagues identified a 37-amino acid peptide, originally termed glucagon-37 and subsequently recognized as OXM [60,63].

Human OXM comprises the complete 29-amino acid sequence of glucagon followed by the C-terminal octapeptide KRNRNNIA [55]. This structural organization preserves the glucagon pharmacophore while modifying receptor recognition sufficiently to generate a pharmacological profile distinct from that of pancreatic glucagon. OXM activates GCGR with lower potency than native glucagon and GLP-1R with lower potency than native GLP-1 [21,22,23]. Its dual activity arises from recognition of the complete peptide by two structurally related Class B1 receptors rather than from a simple assignment of GCGR activity to the glucagon sequence and GLP-1R activity to the C-terminal extension [23,42].

No distinct OXM-specific receptor has been established. Instead, its best-characterized metabolic actions are mediated through GLP-1R and GCGR [21,22,23,64]. This receptor architecture is central to the pharmacological significance of OXM: a single endogenous peptide engages two receptors that later became deliberately combined targets in metabolic drug development.

### 3.2. Biosynthesis, Secretion, and Physiological Context

OXM is generated in enteroendocrine L cells through PC1/3-dependent processing of proglucagon and corresponds to the C-terminal 37-amino acid region of glicentin, conventionally designated glicentin (33–69) [39,40,61]. Because GLP-1 and OXM arise from the same intestinal precursor-processing pathway, both peptides can be released within the coordinated enteroendocrine response to nutrient ingestion [39,40]. Nutrient exposure increases circulating OXM immunoreactivity, consistent with a postprandial secretory pattern [22,61].

Accurate quantification of endogenous OXM remains analytically challenging because OXM, glucagon, and glicentin share overlapping peptide sequences [61,65,66]. Immunoassay cross-reactivity, rapid peptide degradation, sample handling, and the distinction between total immunoreactivity and intact bioactive peptide can substantially influence reported concentrations [65,66]. Mass-spectrometric approaches have strengthened molecular identification of proglucagon-derived peptides in human plasma and provide an important complement to immunochemical measurements [65,66].

The physiological context of OXM differs fundamentally from pharmacological administration. Endogenous OXM is released as one component of a broader postprandial endocrine response, whereas experimental administration exposes GLP-1R and GCGR to concentrations and temporal profiles determined by dose and route of delivery [21,22]. This distinction becomes particularly relevant when endogenous OXM biology is used to inform the design of long-acting co-agonists, whose receptor engagement can persist far beyond the physiological postprandial signal.

### 3.3. Receptor Pharmacology

#### 3.3.1. GLP-1R-Dependent Actions

OXM activates GLP-1R with lower potency than native GLP-1 but retains sufficient receptor activity to produce biologically relevant effects on feeding and glucose regulation [21,22,23]. In receptor-dissection experiments, Baggio and colleagues demonstrated that the acute anorectic response to intracerebroventricular OXM was abolished in GLP-1R-deficient mice but retained in GCGR-deficient mice [23]. These findings identify GLP-1R as a major mediator of the acute anorectic action of OXM.

GLP-1R activation also provides a mechanistic basis for the insulinotropic and glucoregulatory actions of OXM. Studies in humans have demonstrated glucose-lowering activity following administration of native OXM [67], whereas experimental models have shown improvements in β-cell function and glucose regulation [68]. Interestingly, OXM did not reproduce every physiological action associated with native GLP-1 under the conditions examined; in mice, improved glucose regulation occurred without inhibition of gastric emptying [68]. These observations illustrate that engagement of the same receptor does not necessarily generate an identical integrated physiological response when ligand potency, receptor trafficking, concurrent GCGR activation, and exposure differ.

#### 3.3.2. GCGR-Dependent Actions

In parallel with GLP-1R activation, OXM engages GCGR and thereby accesses metabolic pathways associated with hepatic substrate handling and energy expenditure [21,22,23,27]. Experimental studies using OXM analogues have shown that GCGR activation contributes to increases in energy expenditure and to metabolic effects that cannot be reproduced completely by matched GLP-1R activation alone [27,29]. Studies employing receptor-deficient models and pharmacologically matched comparators further supported a contribution of GCGR to body-weight regulation and lipid metabolism within dual-agonist pharmacology [28,29].

These findings provided an important conceptual shift in metabolic pharmacology. GCGR activation had traditionally been viewed predominantly through its capacity to stimulate hepatic glucose production, whereas OXM and subsequently engineered co-agonists demonstrated that GCGR signaling could be incorporated into a broader metabolic intervention when accompanied by sufficient incretin activity [17,18,19,20,28,29]. The resulting therapeutic principle is not unrestricted glucagon action, but controlled GCGR engagement within a receptor context capable of maintaining glycemic control.

#### 3.3.3. Affinity, Potency, Efficacy, and Signaling Context

Affinity, potency, and efficacy describe distinct dimensions of ligand–receptor pharmacology. Affinity reflects ligand binding, potency describes the concentration required to produce a defined response, and efficacy describes the response that a ligand can generate within a specified receptor system [41,52]. Accordingly, the lower potency of OXM relative to GLP-1 at GLP-1R or glucagon at GCGR does not by itself establish partial agonism. Estimates of maximal response depend on receptor density, coupling efficiency, signal amplification, assay duration, and the endpoint measured [23,41,52].

This distinction is particularly relevant for Class B1 GPCRs, for which cAMP generation, β-arrestin recruitment, receptor internalization, recycling, and sustained signaling can display ligand-dependent differences [52,69]. At GLP-1R, OXM can produce robust cAMP responses while exhibiting lower maximal recruitment of β-arrestin and GRK2 than GLP-1 in heterologous systems [70]. Such pathway-dependent differences demonstrate that the pharmacological phenotype of OXM cannot be reduced to a single EC_50_ or Emax value.

More broadly, ligand-specific receptor trafficking provides a mechanism by which acute signaling potency and sustained biological activity may diverge. Studies with engineered GLP-1R agonists have shown that altered receptor internalization and recycling can prolong insulinotropic responses despite reduced acute signaling potency [69]. Although these findings should not be directly assigned to OXM or retatrutide without experimental confirmation, they establish an important design principle for multi-receptor pharmacology: receptor activity is multidimensional and temporally regulated.

### 3.4. Early Human Metabolic Evidence

The translational relevance of OXM was established by early controlled studies in humans. Acute intravenous administration reduced subsequent energy intake, demonstrating that the anorectic activity observed experimentally was preserved in humans [26]. Repeated subcutaneous administration for four weeks subsequently produced a mean body-weight reduction of 2.3 kg, compared with 0.5 kg with placebo [24]. Although modest by contemporary standards, these findings provided early clinical proof that pharmacological exposure to a dual GLP-1R/GCGR agonist could influence human energy balance.

A randomized crossover study further suggested that the metabolic actions of OXM extended beyond suppression of food intake. OXM increased activity-related energy expenditure by approximately 26% and total energy expenditure by approximately 9%, while resting energy expenditure was not significantly increased [25]. The distinction is mechanistically important because it argues against describing the human OXM signal simply as an increase in resting metabolic rate. Together with experimental receptor studies, these findings contributed to the hypothesis that GLP-1R-mediated appetite suppression and GCGR-associated metabolic actions could be combined within a single therapeutic strategy [25,27,28,29].

Native OXM nevertheless differs substantially from contemporary co-agonists in receptor potency, molecular stability, pharmacokinetic exposure, and dosing duration. Its early human studies therefore established biological and translational proof of concept, rather than predicting the magnitude of weight reduction subsequently achieved with engineered long-acting molecules. The principal early human findings are summarized in Table 1.

### 3.5. Pharmacological Limitations and the Transition to Co-Agonists

Despite its informative receptor pharmacology, native OXM has properties that limit its direct therapeutic use. The peptide is rapidly cleared, susceptible to enzymatic degradation, and less potent at GLP-1R and GCGR than their respective cognate ligands [21,22,23,61]. Sustained therapeutic co-activation therefore required molecular engineering rather than simple replacement of the endogenous peptide.

Sequence modification and chemical conjugation provided complementary solutions. Amino acid substitutions increased resistance to proteolysis and allowed relative GLP-1R/GCGR activity to be adjusted, whereas lipidation and other half-life extension strategies prolonged systemic exposure [28,29,71,72]. These modifications transformed receptor co-activation from an endogenous pharmacological observation into a controllable drug-design variable.

The limited development of native OXM as a replacement therapy should therefore not be interpreted as failure of the OXM concept. The concept succeeded through molecular engineering rather than hormone replacement. Engineered GLP-1R/GCGR co-agonists retained the therapeutically attractive principle demonstrated by OXM—simultaneous engagement of complementary receptor systems—while departing substantially from its native sequence, potency ratio, and pharmacokinetic profile [28,29,30,31]. This transition established the pharmacological bridge from endogenous dual agonism to the modern generation of long-acting co-agonists and, ultimately, triagonists such as retatrutide.

## 4. Therapeutic Rationale for Glucagon Receptor Agonism

The therapeutic reappraisal of GCGR signaling emerged from an apparent paradox. Excessive glucagon action contributes to hyperglycemia in T2DM, yet the same receptor regulates hepatic lipid and amino acid metabolism, substrate oxidation, and whole-body energy balance [18,19,20]. Recognition of this broader physiology shifted the pharmacological question from whether glucagon should simply be inhibited to whether selected GCGR-mediated metabolic actions could be harnessed while maintaining glycemic control. This conceptual transition provided the rationale for combining GCGR agonism with incretin receptor activation [16,28,29,30,31].

### 4.1. GCGR Antagonism and Controlled Agonism

The hyperglucagonemia of diabetes and the capacity of glucagon to stimulate hepatic glucose production provided a strong mechanistic rationale for GCGR antagonism [18,73,74]. Pharmacological blockade of GCGR reduced glycemia in clinical studies, confirming that excessive glucagon signaling contributes materially to glucose dysregulation in T2DM [73,74,75]. However, chronic suppression of GCGR also revealed metabolic consequences beyond glucose lowering.

Clinical studies with the small-molecule GCGR antagonist LY2409021 demonstrated this trade-off. Glycemic improvement and elevations in hepatic aminotransferases were reported in phase 2 studies [75]. A separate investigation documented increased hepatic fat content [76], while ambulatory blood-pressure changes were characterized in another clinical study [77]. These findings indicated that sustained GCGR inhibition modifies a broader metabolic network encompassing hepatic lipid handling and systemic physiology, rather than selectively suppressing glucose production.

In parallel, experimental studies increasingly demonstrated that GCGR activation regulates processes potentially advantageous in obesity and metabolic disease, including amino acid catabolism, ureagenesis, fatty acid oxidation, substrate mobilization, and energy expenditure [17,19,20]. This led to a different therapeutic strategy: rather than maximizing or abolishing GCGR signaling, controlled GCGR agonism could be combined with incretin receptor activation to retain catabolic and substrate-mobilizing actions while counterbalancing its hyperglycemic potential [16,28,29,30,31]. The emergence of GLP-1R/GCGR co-agonists therefore represents a pharmacological reconciliation of two apparently opposing observations—glucagon can contribute to diabetic hyperglycemia while simultaneously engaging metabolic pathways of potential therapeutic value.

### 4.2. Hepatic Carbon and Nitrogen Metabolism

The liver is a principal target of glucagon action. GCGR signaling stimulates hepatic glucose production but also coordinates amino acid uptake and catabolism, ureagenesis, fatty acid oxidation, ketogenesis, and broader substrate partitioning [19,20,44]. These actions position glucagon as an endocrine regulator of both carbon and nitrogen metabolism, linking nutrient availability to hepatic substrate utilization.

Amino acid metabolism is particularly important within this framework. The liver–α-cell axis describes a reciprocal relationship in which glucagon promotes hepatic amino acid disposal and ureagenesis, whereas circulating amino acids stimulate pancreatic α-cell glucagon secretion [19,44]. Disruption of hepatic glucagon signaling can therefore alter circulating amino acid concentrations and α-cell function, while metabolic liver disease can modify hepatic responsiveness to glucagon [19,20,44]. This feedback system expands the biological significance of GCGR beyond its classical role in glucose counter-regulation.

GCGR signaling also influences hepatic lipid metabolism. Experimental evidence supports increased fatty acid oxidation and ketogenesis under glucagon action, providing a mechanistic rationale for investigating GCGR agonism in metabolic liver disease [17,20]. Imaging-based reductions in liver fat with retatrutide and survodutide are consistent with this broader metabolic framework [78,79]. Histological MASH and fibrosis endpoints provide a different level of evidence and are evaluated separately [80,81]. However, hepatic responses during treatment arise in parallel with reduced energy intake, weight loss, altered insulin action, and changes in substrate delivery, making the net phenotype an integrated consequence of multiple pathways.

This distinction is particularly relevant when interpreting changes in adiposity. Hepatic fatty acid oxidation and whole-body negative energy balance can contribute to loss of adipose mass without requiring a major direct lipolytic action of glucagon on human adipocytes [20,82]. Tissue-specific receptor expression and substrate flux must therefore be considered when linking systemic weight loss to cellular GCGR mechanisms.

### 4.3. Energy Expenditure and Metabolic Adaptation

The potential contribution of GCGR signaling to energy expenditure represents one of the most attractive—and mechanistically unresolved—aspects of glucagon-containing polyagonism. Experimental glucagon pharmacology, OXM analogues, and engineered GLP-1R/GCGR co-agonists have demonstrated increases in energy expenditure under selected conditions, particularly in rodent models [17,27,28,83]. Early human studies with native OXM also identified increased activity-related and total energy expenditure, although resting energy expenditure was not significantly increased [25]. Together, these findings provide biological plausibility for an energetic component of GCGR-containing pharmacology.

Interpretation becomes more complex during substantial weight loss. Total energy expenditure normally declines as body mass decreases, reflecting reductions in metabolically active tissue as well as adaptive thermogenesis [5,6]. Consequently, a pharmacological effect on energy expenditure need not appear as an absolute increase above pretreatment values. Attenuation of the decline expected for a given change in fat-free mass, fat mass, energy intake, and physical activity could also represent a biologically relevant effect. Longitudinal studies should therefore distinguish absolute expenditure from expenditure adjusted for changing body composition and should measure physical activity and energy intake concurrently.

The mechanistic link between GCGR activation and human energy expenditure remains incompletely resolved. Candidate pathways include hepatic substrate cycling, sympathetic and neuroendocrine responses, altered nutrient oxidation, and endocrine mediators induced downstream of glucagon signaling [17,20,83]. Their relative contribution is likely to depend on species, dose, duration of exposure, nutritional state, and the accompanying activity of GLP-1R and GIPR.

FGF21 illustrates the importance of testing these mechanisms directly. Although glucagon can influence hepatic FGF21 biology experimentally, circulating FGF21 decreased at several retatrutide doses in the MASLD substudy while liver fat declined markedly [78]. This dissociation argues against a simple model in which sustained elevation of circulating FGF21 is required to explain the hepatic response to retatrutide. More broadly, the magnitude of weight loss produced by a GCGR-containing polyagonist cannot by itself quantify the contribution of increased energy expenditure or any individual GCGR-dependent pathway.

## 5. Molecular Engineering and Clinical Differentiation of GLP-1R/GCGR Co-Agonists

The transition from native OXM to engineered GLP-1R/GCGR co-agonists transformed dual receptor activation from an endogenous observation into a controllable pharmacological strategy. Medicinal chemistry enabled receptor activity, proteolytic stability, and pharmacokinetic exposure to be tuned through complementary molecular modifications, allowing investigators to test whether GCGR engagement could add metabolic effects to GLP-1R agonism while preserving glycemic control [28,29,41]. This principle subsequently generated several clinical candidates that share GLP-1R/GCGR agonism but differ substantially in molecular architecture, receptor pharmacology, dosing, and therapeutic development.

### 5.1. Molecular Design Principles and Preclinical Proof of Concept

Early synthetic co-agonist studies established that GLP-1R/GCGR pharmacology could be deliberately engineered and experimentally dissected. Day and colleagues developed a glucagon-based peptide with combined GLP-1R and GCGR activity that improved body weight, adiposity, and glucose metabolism in rodent models [28]. Pocai and colleagues subsequently used pharmacologically matched peptide comparators and receptor-deficient models to distinguish the contribution of GCGR activity from GLP-1R agonism alone [29]. Together, these studies provided mechanistic proof of concept that simultaneous activation of GLP-1R and GCGR could generate metabolic effects beyond those obtained with the corresponding GLP-1R-selective comparator [28,29].

A central insight from these experiments was that receptor balance is an engineered property rather than a fixed characteristic of dual agonism. Amino acid substitutions can simultaneously alter proteolytic stability, receptor affinity, potency, intrinsic efficacy, and signaling behavior, whereas lipidation and other conjugation strategies modify albumin association, clearance, and duration of systemic exposure [28,29,41]. Consequently, two peptides with apparently similar GLP-1R:GCGR potency relationships in vitro may generate different pharmacological phenotypes in vivo if their exposure profiles, receptor efficacy, or tissue accessibility differ.

Matched comparator molecules therefore became particularly informative for mechanistic studies. By maintaining similar GLP-1R activity and pharmacokinetic properties while modifying GCGR engagement, such designs can more directly interrogate the incremental contribution of glucagon receptor signaling [29]. This experimental logic established the foundation for subsequent clinical co-agonists, in which receptor pharmacology and half-life extension were optimized simultaneously rather than sequentially.

#### 5.1.1. Proteolytic Stability and Half-Life Extension

Protection from enzymatic cleavage and prolongation of systemic exposure are related but distinct design tasks. Noncanonical residues at susceptible positions can reduce proteolysis, whereas fatty-acid or surfactant conjugation can increase albumin association and extend exposure. The palmitoyl modification of cotadutide, C18-diacid modification of survodutide, and surfactant conjugation of pemvidutide illustrate different implementations [84,85,86]. Conjugation site, linker, and lipid chemistry must be optimized together because they can also alter receptor access and functional potency. A prolonged circulating half-life therefore does not establish sustained or equivalent engagement of every target receptor.

#### 5.1.2. Activity-Ratio Design

Activity-ratio design seeks a clinically useful combination of incretin-mediated glucose control and GCGR-mediated substrate mobilization. Concentration–response curves at each receptor should be interpreted with their maximal responses, assay conditions, and cognate reference ligands. Matched comparator peptides can help isolate the effect of changing GCGR activity while maintaining the remaining pharmacology and exposure as closely as possible [28,29]. Optimization should include pharmacokinetics and glycemic safety rather than selecting a ratio from cAMP potency alone. The preferred ratio may differ across therapeutic objectives and metabolic states; no universal optimum has been established.

#### 5.1.3. Receptor Selectivity Determinants

The conserved Class B1 binding architecture creates opportunities for shared recognition, but selectivity depends on receptor-specific contacts involving the extracellular domain, extracellular loops, and transmembrane pocket. Structural and mutagenesis studies of dual agonists identify interactions that can be adjusted through peptide substitutions [42]. Because the same residue can contribute differently at different receptors, improving activity at one target may compromise another. Candidate substitutions therefore require reassessment across the complete target panel; a static structure or a favorable contact at one receptor cannot establish the integrated selectivity profile.

#### 5.1.4. Signaling Bias and Receptor Trafficking

Engineering can also modify the relationship between cAMP signaling, β-arrestin recruitment, internalization, and recycling. Experiments with GLP-1R agonists show that altered trafficking can sustain insulinotropic responses despite lower acute potency [69]. This is a design rationale, not proof that a particular bias profile improves the clinical benefit–risk balance of every dual agonist. Claims of bias require comparison with a reference agonist across pathways under matched experimental conditions. Receptor- and time-dependent measurements are needed to distinguish pathway preference from differences in receptor reserve, assay amplification, or exposure.

### 5.2. Pharmacological Differentiation of Clinical GLP-1R/GCGR Co-Agonists

Cotadutide (MEDI0382; AstraZeneca, Cambridge, UK) provided early clinical proof of concept for sustained GLP-1R/GCGR co-agonism in humans. Developed as a once-daily subcutaneous peptide, its clinical program examined not only body weight and glycemic control but also mixed-meal glucose responses, insulin secretion, gastric emptying, and hepatic metabolism [87,88]. In the phase 2a study reported by Ambery and colleagues, cotadutide reduced postprandial glucose exposure and body weight relative to placebo [88]. The program is particularly informative mechanistically because it incorporated controlled nutrient challenges and organ-level metabolic measurements early in clinical development.

Earlier hepatic evidence was also reported under NAFLD/NASH terminology. Cotadutide improved metabolic and liver-related biochemical or noninvasive measures in a 54-week phase 2b trial, but the exploratory hepatic analyses did not establish histological fibrosis regression [89]. In experimental NASH models, cotadutide improved hepatic lipid handling and histological disease through complementary GLP-1R and GCGR actions [90]. These findings support the translational rationale while requiring clear separation of animal histology, human noninvasive markers, and biopsy-confirmed clinical outcomes.

Mazdutide (IBI362/LY3305677; Innovent Biologics, Suzhou, China) represents a distinct development strategy based on a long-acting OXM analogue with GLP-1R/GCGR co-agonist activity. Its clinical program progressed to phase 3 evaluation in obesity, providing large-scale evidence that dual receptor agonism can support substantial and sustained weight reduction [91,92]. In GLORY-1, mean body-weight changes at week 48 under the treatment-policy estimand were −11.00% with 4 mg and −14.01% with 6 mg, compared with +0.30% with placebo [91]. GLORY-2 subsequently extended the program to the 9-mg dose in a distinct phase 3 population [92]. Together, these studies position mazdutide as an advanced clinical implementation of GLP-1R/GCGR co-agonism in obesity and metabolic disease. The disclosed sequence contains Aib2 and a Lys20-linked (AEEA)_2_–γGlu–C20 fatty-diacid side chain, with C-terminal amidation [93]. In a separate high-dose phase 1 study, the terminal half-life at 16 mg was approximately eight days at week 20; this estimate is specific to that regimen and should not be treated as a dose-independent constant [94].

Survodutide (BI 456906; Boehringer Ingelheim, Ingelheim am Rhein, Germany; developed in collaboration with Zealand Pharma, Søborg, Denmark) has followed a different translational trajectory, with parallel development in obesity and metabolic liver disease. In biopsy-characterized MASH, phase 2 evaluation demonstrated histological improvement of MASH without worsening of fibrosis [80]. Its obesity program subsequently progressed to phase 3: in SYNCHRONIZE-1, mean body-weight changes at week 76 under the treatment-regimen estimand were −12.2% and −13.0% with 3.6 and 6.0 mg, respectively, compared with −5.4% with placebo [95]. This combination of obesity and histological liver-disease development makes survodutide particularly informative for evaluating the metabolic and hepatic consequences of sustained GLP-1R/GCGR co-agonism.

SYNCHRONIZE-2 extended phase 3 evaluation to 752 adults with T2DM and BMI ≥ 27 kg/m^2^ (NCT06066528). At week 76, mean body-weight changes under the treatment-regimen estimand were −8.2% and −9.8% with once-weekly survodutide 3.6 and 6.0 mg, respectively, versus −3.9% with placebo. Gastrointestinal adverse events occurred in 72.8%, 77.7%, and 38.6% of participants, respectively [96]. These findings provide direct evidence in T2DM, but comparison with SYNCHRONIZE-1 does not establish a causal effect of diabetes status on treatment response.

Pemvidutide (ALT-801; Altimmune, Gaithersburg, MD, USA) provides another pharmacologically distinct implementation of GLP-1R/GCGR co-agonism and has been developed with particular emphasis on obesity and MASH. In the IMPACT trial, treatment increased the proportion of participants achieving MASH resolution without worsening of fibrosis at 24 weeks, whereas the coprimary endpoint of fibrosis improvement was not met [81]. This divergence between steatohepatitis resolution and fibrosis response illustrates that hepatic outcomes within the same co-agonist program may evolve on different biological and temporal scales. Rapid depletion of intrahepatic lipid and resolution of inflammatory injury can precede the slower turnover and remodeling of fibrotic extracellular matrix; fibrosis regression may remain incomplete or absent. Thus, even a relative liver-fat reduction of ≥80% by MRI-PDFF cannot be interpreted as complete MASH resolution or stage-specific fibrosis regression [78,80,81].

Importantly, these molecules represent distinct implementations of a shared receptor strategy rather than interchangeable members of a homogeneous pharmacological class. They differ in peptide sequence, relative GLP-1R/GCGR activity, half-life-extension strategy, dosing schedule, clinical populations, and development priorities [41,80,81,84,85,86,87,88,91,92,95]. Cotadutide incorporates a palmitoyl-based conjugation strategy, survodutide uses a C18 diacid-containing modification, and pemvidutide employs a glycolipid-surfactant conjugation platform [84,85,86]. Mazdutide is a long-acting OXM analogue with a distinct molecular design [91,92]. These differences provide a natural experiment in how receptor pharmacology and pharmacokinetic engineering can generate divergent clinical development trajectories despite a shared GLP-1R/GCGR target pair (Figure 2; Table 2).

### 5.3. Translational Lessons from Dual-Agonist Development

Clinical development of GLP-1R/GCGR co-agonists established that pharmacologically meaningful GCGR activity can coexist with improved glycemic control when integrated with GLP-1R agonism [80,81,87,88,91,92,95]. Across development programs, dual agonism has been associated with reductions in body weight and improvements in metabolic liver phenotypes, providing human translational support for the principle initially suggested by OXM and engineered preclinical co-agonists [28,29,80,81,87,88,91,92,95].

Importantly, the diversity of these programs demonstrates that receptor combination alone does not define the clinical phenotype. Cotadutide emphasized early metabolic physiology, mazdutide progressed to large phase 3 obesity trials, survodutide combined advanced obesity development with histological MASH evaluation, and pemvidutide generated a distinct obesity–liver development program [80,81,87,88,91,92,95]. Their differences in sequence, relative receptor activity, molecular modification, pharmacokinetic exposure, dose, and clinical population represent multiple implementations of the same receptor pair rather than a uniform GLP-1R/GCGR class effect.

A second lesson concerns receptor balance. Descriptors such as “balanced”, “GLP-1R-preferential”, or “GCGR-preferential” are meaningful only when anchored to a defined assay system, reference ligand, and exposure profile [31,41]. Relative potency measured in vitro does not directly specify relative receptor contribution in vivo, and a prominent hepatic or weight phenotype does not itself establish tissue selectivity. The clinically expressed phenotype instead emerges from the interaction between receptor pharmacology, pharmacokinetic exposure, and biological context.

Dual-agonist programs and preclinical triagonist studies support related pharmacological strategies rather than a strictly sequential clinical lineage. The experimental incorporation of GIPR activity into a GLP-1R/GCGR-active peptide preceded the clinical development of retatrutide [30,31]. The relevant design question is whether simultaneous GIPR, GLP-1R, and GCGR activation can integrate insulinotropic, anorectic, and substrate-mobilizing pathways within a single molecule [30,31].

## 6. Pharmacological and Structural Basis of Retatrutide Triagonism

The transition from GLP-1R/GCGR co-agonism to triple GIPR/GLP-1R/GCGR agonism introduced a third component into an already integrated pharmacological system. GIPR was particularly attractive because of its established insulinotropic activity, its metabolic actions beyond the pancreatic β-cell, and the clinical validation of sustained GIPR/GLP-1R co-agonism [11,12,56]. Preclinical triagonists subsequently demonstrated that agonist activity at GIPR, GLP-1R, and GCGR could be incorporated within a single peptide and produce substantial metabolic effects [30,99,100]. Retatrutide translated this principle into a long-acting molecule designed for sustained activation of all three receptors [31].

### 6.1. Rationale for Incorporating GIPR

Early unimolecular dual incretins and experimental triagonists established that GIPR activity could be combined with GLP-1R and GCGR agonism within a single peptide scaffold [30,99,100]. The rationally designed GIPR/GLP-1R/GCGR triagonist reported by Finan and colleagues produced greater reductions in body weight and improvements in glucose and lipid metabolism than comparator interventions in rodent models, providing preclinical proof of concept for triple receptor agonism [30]. These experiments established the feasibility of the receptor combination before retatrutide entered clinical development.

GIPR adds pharmacological actions that are complementary to, but not redundant with, GLP-1R and GCGR signaling. In pancreatic β-cells, GIPR activation enhances glucose-dependent insulin secretion, providing a mechanism that can support glycemic control during simultaneous GCGR activation [37,56]. GIPR signaling also operates in neuronal and adipose compartments implicated in feeding behavior, nutrient handling, and energy balance [56,57,58,59]. Within a triagonist, GIPR activity can therefore contribute to an integrated phenotype involving insulin secretion, appetite regulation, nutrient partitioning, and metabolic adaptation.

The relative contribution of GIPR is expected to vary with biological context. Glucose concentration, β-cell functional reserve, insulin sensitivity, receptor expression, and concurrent GLP-1R and GCGR activity can all influence the integrated response [31,56,101,102]. Triple agonism is therefore better understood as coordinated receptor pharmacology than as the addition of a single GIPR-mediated “glycemic shield” to a GLP-1R/GCGR co-agonist.

### 6.2. The GIPR Agonism–Antagonism Paradox

The pharmacology of GIPR presents an apparent paradox: both receptor agonism and receptor antagonism can enhance weight reduction when combined with GLP-1R-active pharmacology [57,58,59,103]. Tirzepatide provides clinical validation of sustained GIPR/GLP-1R agonism [11,12], whereas maridebart cafraglutide combines GIPR antagonism with GLP-1R agonism and has produced substantial weight reduction in clinical development [103]. These observations indicate that opposite modes of GIPR modulation can converge on favorable energy-balance phenotypes through mechanistically distinct pathways.

One proposed mechanism involves exposure-dependent receptor desensitization. Sustained GIPR agonism can attenuate subsequent receptor responsiveness in adipocyte systems, producing a state that shares some functional features with receptor antagonism [58]. This mechanism provides a potential explanation for convergence under prolonged exposure, but GIPR regulation is cell-type dependent. Receptor desensitization in adipocytes does not imply equivalent signaling adaptation in pancreatic β-cells or GIPR-expressing neuronal populations.

Neural circuit studies provide evidence for an additional explanation based on anatomical separation of GIPR function. In mice, GIPR agonism can suppress food intake through GIPR-expressing GABAergic neurons, whereas the weight-lowering effects of GIPR antagonism depend more strongly on concurrent GLP-1R signaling [57]. Regional receptor-disruption experiments further implicate area-postrema GIPR in anorectic responses to agonism and hypothalamic GIPR in the ability of antagonism to enhance GLP-1R-associated weight reduction [59]. Thus, agonism and antagonism may converge on body-weight reduction by engaging different cellular populations and circuit-level mechanisms rather than by producing pharmacologically equivalent receptor states.

Selective GIPR agonism provides an additional experimental test of this biology. LY3537021 (Eli Lilly and Company, Indianapolis, IN, USA) has undergone preclinical characterization and phase 1 evaluation in healthy participants and individuals with T2DM [104]. Short-term administration produced evidence of pharmacological activity, including reductions in body weight, demonstrating that GIPR agonism can generate measurable metabolic effects independently of GLP-1R co-agonism [104]. However, the fasting-glucose reductions in participants with T2DM were transient and were not significantly different from placebo at day 29 [104]. Whether selective GIPR agonism can produce durable obesity treatment comparable with multi-receptor approaches remains an open clinical question.

Together, these findings support a model in which the metabolic consequences of GIPR modulation depend on direction of receptor modulation, duration of exposure, cellular localization, and concurrent receptor signaling [57,58,59,103,104]. This context dependence provides a mechanistic bridge to the Activity–Exposure–Context framework developed later in this review and argues for direct physiological characterization rather than classification based solely on circulating GIP concentrations.

### 6.3. Molecular Architecture, Receptor Activity, and Pharmacokinetic Exposure

Retatrutide (LY3437943; Eli Lilly and Company, Indianapolis, IN, USA) is a 39-amino acid, GIP-based peptide engineered to activate GIPR, GLP-1R, and GCGR within a single long-acting molecular scaffold [31,43]. Its sequence incorporates the noncanonical amino acid α-aminoisobutyric acid (Aib) at positions 2 and 20, α-methyl-L-leucine at position 13, and a C-terminal amide [31,43]. A C20 fatty-diacid moiety is conjugated through the side chain of Lys17, promoting albumin association and markedly prolonging systemic exposure [31,43]. The resulting pharmacokinetic profile supports once-weekly administration, with a clinical elimination half-life of approximately six days [31,101]. The molecular architecture of retatrutide, including its sequence modifications, lipid-conjugation site, and relative receptor activities, is summarized in Figure 3.

Retatrutide was not engineered to reproduce the potency of the three endogenous hormones equally. In functional assays used during its pharmacological characterization, retatrutide displayed approximately 8.9-fold the potency of native GIP at GIPR, 0.4-fold the potency of GLP-1 at GLP-1R, and 0.3-fold the potency of glucagon at GCGR [31,43]. Because each value is normalized to a different cognate ligand, these ratios describe receptor-specific functional potency within the corresponding assay rather than a common quantitative scale of receptor engagement. In particular, in vitro EC_50_ ratios do not translate linearly into in vivo fractional receptor occupancy or clinical efficacy. EC_50_ is a functional assay parameter rather than a binding dissociation constant, and receptor occupancy cannot be calculated from native-ligand-normalized potency ratios alone. In vivo receptor contribution additionally depends on intrinsic efficacy, receptor abundance, tissue accessibility, drug exposure, and signaling kinetics. Thus, the pharmacological identity of retatrutide is defined by simultaneous but quantitatively distinct activity at three related receptors under sustained systemic exposure (Figure 3).

### 6.4. Structural Basis of Triple Receptor Recognition

Cryo-electron microscopy has resolved retatrutide bound to Gs-coupled GLP-1R, GIPR, and GCGR, providing a structural explanation for how a single peptide can activate three related Class B1 GPCRs [43]. Across the three complexes, the N-terminal region of retatrutide penetrates the transmembrane receptor core and participates in activation, whereas the more C-terminal helical region engages the extracellular domain and extracellular receptor surfaces (Figure 3) [43]. This overall architecture is consistent with the conserved two-domain recognition mechanism characteristic of peptide-activated Class B1 GPCRs.

Despite this shared binding mode, retatrutide is accommodated differently by each receptor. Variations in extracellular-loop geometry and residues within the extracellular ends of the transmembrane helices generate receptor-specific interaction networks superimposed on the conserved binding architecture [43]. Structure–function and mutagenesis studies identified receptor-specific determinants that modify retatrutide potency, demonstrating that triple agonism emerges from shared molecular recognition combined with receptor-specific accommodation rather than from three physically separable pharmacophores within the peptide [43].

This distinction has direct implications for peptide engineering. Sequence substitutions that improve interaction with one receptor can simultaneously alter recognition by the other two, making receptor balance an emergent property of the complete peptide–receptor interface [31,43]. The structural data therefore provide a molecular basis for rationally tuning multi-receptor activity, but functional characterization remains necessary to determine how individual contacts influence potency, efficacy, signaling kinetics, and receptor trafficking.

Cryo-EM structures represent stabilized receptor conformations and should be integrated with dynamic pharmacological measurements. Available retatrutide structures predominantly resolve Gs-coupled active states [43]; β-arrestin recruitment, internalization, recycling, endosomal signaling, and long-term receptor regulation require complementary functional experiments [42,52,69]. This distinction is particularly important when comparing retatrutide with GLP-1R/GCGR co-agonists whose signaling and trafficking profiles may differ despite overlapping receptor targets.

## 7. Clinical Development of Retatrutide

The clinical development of retatrutide has progressed from early pharmacokinetic and dose-escalation studies to randomized phase 2 and phase 3 trials encompassing T2DM, obesity, and metabolic liver disease. Across these studies, escalating receptor exposure has been associated with dose-dependent effects on glycemic control, body weight, and cardiometabolic phenotypes, providing clinical translation of the triple-agonist pharmacology described above [78,101,102,105,106,107,108,109,110]. The rationale and design of the TRIUMPH registrational program have been reported separately from trial outcomes [111].

### 7.1. Early Clinical Development and Phase 2 Proof of Concept

First-in-human and phase 1b studies established the pharmacokinetic profile, initial tolerability, and metabolic activity of retatrutide, supporting once-weekly administration and subsequent dose-ranging development [31,101]. In people with T2DM, the randomized phase 2 trial compared retatrutide with placebo and dulaglutide and demonstrated substantial dose-dependent improvements in glycemic control and body weight [105]. At the highest evaluated dose, HbA1c decreased by approximately 2.02 percentage points at week 24, while body weight decreased by approximately 16.94% at week 36 [105]. These findings provided early clinical evidence that simultaneous GIPR/GLP-1R/GCGR agonism could achieve substantial glycemic and weight effects within the same therapeutic program.

The phase 2 obesity trial extended these observations to adults without diabetes; its primary weight endpoint was assessed at week 24 [106]. At the later 48-week assessment, participants receiving 12 mg retatrutide achieved a mean body-weight reduction of 24.2%, compared with 2.1% with placebo [106]. Weight reduction was dose dependent and continued throughout much of the observation period, establishing a strong clinical efficacy signal and providing the rationale for the subsequent TRIUMPH phase 3 obesity program [106].

Retatrutide also produced marked changes in hepatic steatosis. In the phase 2 MASLD substudy, which included 98 participants with baseline liver fat ≥ 10%, treatment with 12 mg produced a mean relative reduction in liver fat of 82.4% at week 24, compared with a 0.3% increase with placebo, as assessed by MRI-PDFF [78]. This finding established a substantial imaging-based hepatic response and strengthened interest in glucagon-containing polyagonism for metabolic liver disease [78]. MRI-PDFF quantifies hepatic steatosis rather than histological MASH resolution or fibrosis, preserving an important distinction between imaging and histological liver endpoints. Accordingly, a relative MRI-PDFF reduction of ≥80%, or even normalization of imaging-defined liver fat, does not establish complete MASH resolution or fibrosis regression at any particular stage. The time course of lipid clearance can differ from that of inflammatory resolution and extracellular-matrix remodeling, requiring endpoint-specific longitudinal assessment [80,81].

### 7.2. Phase 3 Efficacy in Type 2 Diabetes

Phase 3 development in T2DM addressed a central pharmacological question for retatrutide: whether sustained GCGR agonism could be incorporated into a GIPR/GLP-1R/GCGR triagonist while preserving robust glycemic control. TRANSCEND-T2D-1 provided the first phase 3 evidence in people with relatively early T2DM inadequately controlled with diet and exercise, demonstrating dose-dependent reductions in HbA1c, fasting glucose, and body weight [102]. The trial randomized 537 participants. At week 40, retatrutide 12 mg reduced HbA1c by 1.94 percentage points and body weight by 15.3%, compared with reductions of 0.81 percentage points and 2.6%, respectively, with placebo under the treatment-regimen estimand [102]. These findings established that pharmacologically meaningful GCGR activity can coexist with substantial glucose lowering when integrated with simultaneous GIPR and GLP-1R agonism.

TRIUMPH-2 extended phase 3 evaluation to 1152 adults with obesity or overweight and T2DM treated for 80 weeks [109]. Under the treatment-regimen estimand, mean body-weight changes were −11.9%, −16.8%, and −18.8% with retatrutide 4, 9, and 12 mg, respectively, compared with −5.1% with placebo [109]. Under the efficacy estimand, the corresponding reductions were −12.7%, −19.1%, and −20.8%, compared with −4.0% with placebo [109,112]. In the reported primary analysis, mean HbA1c changes were −1.38, −1.50, and −1.45 percentage points with retatrutide 4, 9, and 12 mg, respectively, compared with −0.45 percentage points with placebo [109]. The efficacy estimand addresses a hypothetical scenario in which randomized participants remained on study intervention, with allowed dose interruptions or modifications, without initiating prohibited weight-management treatment; glycemic rescue is additionally addressed for glycemic endpoints [112]. It is not an analysis restricted to observed treatment completers and should be distinguished from the treatment-regimen estimand.

Together, TRANSCEND-T2D-1 and TRIUMPH-2 demonstrate that substantial weight reduction and glycemic improvement can coexist during sustained triple-receptor agonism in T2DM [102,109]. This finding is mechanistically relevant because GCGR activation is intrinsically capable of increasing hepatic glucose production, whereas the integrated pharmacology of retatrutide produces a net glucose-lowering phenotype. The clinical data therefore support the principle that the metabolic consequences of GCGR agonism depend on its incorporation within a broader receptor and exposure context rather than on GCGR activation in isolation.

The two trials also represent different metabolic settings. TRANSCEND-T2D-1 examined relatively early T2DM managed with diet and exercise, whereas TRIUMPH-2 evaluated participants with both excess adiposity and established T2DM [102,109]. This distinction is relevant to multi-receptor pharmacology because β-cell reserve, insulin sensitivity, adiposity, and disease duration may influence the integrated response to simultaneous GIPR, GLP-1R, and GCGR activation. These variables provide a clinical rationale for the Activity–Exposure–Context framework developed later in this review.

### 7.3. Phase 3 Efficacy in Obesity and Obesity-Related Complications

The TRIUMPH program extended retatrutide development from metabolic proof of concept to long-term treatment of obesity and its associated complications. TRIUMPH-1 randomized 2339 adults with obesity, or overweight with at least one weight-related complication, without diabetes to placebo or retatrutide 4, 9, or 12 mg and evaluated body-weight change over 80 weeks [108]. Under the treatment-regimen estimand, mean body-weight changes were −17.6%, −23.7%, and −25.0%, respectively, compared with −3.9% with placebo [108]. Under the efficacy estimand, the corresponding reductions were −19.0%, −25.9%, and −28.3%, compared with −2.2% with placebo [107,108]. At the 12-mg dose, 45.3% of participants achieved ≥30% weight reduction under the efficacy estimand, illustrating the depth of response observed within the trial [107,108].

A prespecified extension of TRIUMPH-1 evaluated continued treatment through week 104 in a subset of participants with baseline BMI ≥ 35 kg/m^2^. Under the efficacy estimand reported by the sponsor, mean body-weight reductions reached −27.9%, −29.5%, and −30.3% across the 4-mg-to-maximum-tolerated-dose, 9-mg-to-maximum-tolerated-dose, and 12-mg-to-maximum-tolerated-dose strategies, respectively [107]. These results describe a selected extension population that completed the main study and tolerated the assigned dose; they should not be generalized to all participants originally randomized or conflated with the primary 80-week analysis [107].

The phase 3 program has also evaluated retatrutide in populations in which obesity coexists with clinically important complications. In sponsor-reported TRIUMPH-3 results, adults with severe obesity and established cardiovascular disease, with or without T2DM, achieved mean body-weight reductions of −21.6% and −22.6% with retatrutide 9 and 12 mg, respectively, compared with −3.2% with placebo at week 80 under the efficacy estimand [113]. The trial also documented substantial changes in several cardiovascular risk markers, including triglycerides, non-HDL cholesterol, systolic blood pressure, waist circumference, and high-sensitivity C-reactive protein [113]. However, the observed MACE analyses were not powered to establish cardiovascular-event reduction; for MACE-3, the reported in-study hazard ratio was 1.12 (95% CI 0.64–1.96) [113]. Thus, TRIUMPH-3 presently provides evidence of weight and cardiometabolic risk-factor modification rather than demonstrated cardiovascular-outcome benefit.

Sponsor-reported TRIUMPH-4 results concern adults with obesity or overweight and knee osteoarthritis without diabetes [110]. At week 68, mean body-weight reductions under the efficacy estimand were −26.4% with 9 mg and −28.7% with 12 mg, compared with −2.1% with placebo [110]. Retatrutide simultaneously produced substantial improvements in WOMAC pain and physical function; mean WOMAC pain scores decreased by 4.5 and 4.4 points with 9 and 12 mg, respectively, compared with 2.4 points with placebo [110]. These findings extend the clinical phenotype of retatrutide beyond body-weight reduction to an obesity-associated functional complication in which mechanical loading, mobility, inflammation, and weight loss may interact.

Across the TRIUMPH program, the magnitude of weight reduction varies according to population and clinical context. Weight loss has been numerically greater in trials enrolling participants without T2DM than in TRIUMPH-2, which enrolled participants with T2DM [107,108,109,110,113]. This pattern is consistent with observations from other incretin-based obesity therapies, but the TRIUMPH trials were not designed as randomized comparisons between diabetic and non-diabetic metabolic states. The cross-trial difference therefore provides a hypothesis-generating example of how baseline metabolic context may influence the phenotypic expression of the same multi-receptor intervention rather than a quantitative estimate of a diabetes-specific modifier of treatment efficacy.

The progression from phase 2 proof of concept to phase 3 efficacy across T2DM, obesity, and obesity-associated complications, together with the principal glycemic and body-weight outcomes of TRANSCEND-T2D-1 and the TRIUMPH program, is summarized in Figure 4 and Table 3.

### 7.4. Tolerability and Safety

The substantial efficacy of retatrutide is accompanied by a tolerability profile dominated by gastrointestinal adverse events, with their frequency generally increasing at higher doses. In TRIUMPH-1, nausea occurred in 28.6%, 38.4%, and 42.4% of participants receiving retatrutide 4, 9, and 12 mg, respectively, compared with 14.8% with placebo [107,108]. Discontinuation because of adverse events occurred in 4.1%, 6.9%, and 11.3%, respectively, compared with 4.9% with placebo [107,108]. These findings make dose escalation, achieved dose, and treatment persistence important components of the clinical interpretation of retatrutide efficacy.

Dysesthesia emerged as a notable adverse event in TRIUMPH-1, occurring in 5.1%, 12.3%, and 12.5% of participants receiving 4, 9, and 12 mg, respectively, compared with 0.9% with placebo [107,108]. Urinary tract infections were reported in 7.5%, 8.8%, and 8.4% of the corresponding retatrutide groups compared with 5.3% with placebo [107,108]. The biological basis and reproducibility of these signals across populations require continued characterization within the broader phase 3 program.

The magnitude of weight reduction achieved with retatrutide also increases the importance of evaluating body composition, nutritional adequacy, and physical function. Glucagon biology provides an additional mechanistic rationale for this assessment because GCGR signaling participates in hepatic amino acid uptake, catabolism, and ureagenesis [19,44]. Longitudinal evaluation of fat mass, lean mass, dietary protein intake, muscle strength, physical performance, and circulating amino acid profiles will therefore be important for defining the qualitative composition of profound pharmacological weight loss rather than relying on total body weight alone.

A phase 2 DXA substudy in T2DM documented fat-mass reduction with retatrutide and a proportion of lean-mass loss broadly consistent with other weight-loss interventions [114]. This does not establish preservation of contractile muscle or physical function, because DXA-derived lean mass includes nonmuscle tissues and is influenced by hydration. Future protocols should prespecify baseline and repeated assessments during dose escalation, active weight loss, and weight maintenance, combining body composition with standardized handgrip dynamometry, chair-rise testing, and gait speed or a short physical performance battery. Dietary protein and energy intake, resistance exercise, age, and baseline frailty should be recorded to contextualize change. Nitrogen balance is best examined in a mechanistic substudy using quantified nitrogen intake and timed urinary nitrogen collection, with allowance for nonurinary losses and collection completeness. If urinary urea nitrogen is used, nonurea losses must also be estimated. Renal function, hepatic urea synthesis, and changing intake complicate interpretation; circulating urea or amino acids alone cannot establish nitrogen balance or muscle catabolism. This tiered approach would test whether profound weight loss preserves function rather than infer functional safety from body weight or lean mass alone [19,44].

Safety interpretation must also distinguish cardiometabolic risk-factor modification from clinical outcome evidence. Retatrutide has produced substantial reductions in body weight and improvements in glycemia, blood pressure, circulating lipids, waist circumference, inflammatory markers, and hepatic steatosis across phase 2 and phase 3 studies [78,102,108,109,110,113]. These changes are clinically relevant, but cardiovascular, renal, and liver-event benefits require appropriately powered outcome trials. The sponsor-reported MACE analyses from TRIUMPH-3 illustrate this distinction: confidence intervals were wide and did not establish a cardiovascular-event benefit despite marked improvements in several cardiovascular risk factors [113]. Finally, the long-term benefit–risk profile of triple-receptor agonism will depend not only on maximal efficacy but also on treatment persistence, dose tolerability, nutritional and functional consequences of large weight reductions, and outcomes during prolonged exposure. These considerations are particularly relevant for chronic obesity treatment, in which therapeutic benefit is expected to extend over years rather than the duration of a registration trial.

## 8. Receptor-Specific Causal Attribution in Multi-Receptor Pharmacology

Clinical trials establish the therapeutic effects of a multi-receptor agonist as an integrated molecule, whereas mechanistic pharmacology seeks to determine how individual receptor components contribute to those effects. This distinction becomes particularly important for retatrutide because GIPR, GLP-1R, and GCGR regulate overlapping but non-identical aspects of insulin secretion, appetite, hepatic substrate metabolism, and energy balance [19,20,31]. The magnitude of the clinical response therefore defines the efficacy of triple agonism but does not, by itself, quantify the contribution of each receptor. Resolving this causal attribution gap requires experimental designs that perturb individual receptor components while preserving the remaining pharmacology as closely as possible.

### 8.1. Whole-Drug Efficacy and Receptor-Specific Mechanisms

Randomized clinical trials of retatrutide estimate the treatment effect of the complete GIPR/GLP-1R/GCGR triagonist under the exposure conditions achieved in the study population [78,102,105,106,107,108,109,110,113]. Because the three receptor activities are delivered simultaneously within the same molecular scaffold, conventional placebo-controlled trials cannot decompose the observed reduction in body weight, HbA1c, or liver fat into independent GIPR-, GLP-1R-, and GCGR-mediated components. Cross-trial comparisons with selective GLP-1R agonists or GIPR/GLP-1R co-agonists face the same limitation because molecular structure, receptor pharmacology, dose, pharmacokinetic exposure, population, treatment duration, and statistical estimands differ across development programs [7,8,9,10,11,12].

Preclinical co-agonist studies provide a useful experimental model for receptor-specific attribution. Pocai and colleagues compared peptides designed to retain similar GLP-1R activity and pharmacokinetic properties while modifying GCGR activity, and receptor-deficient models were used to test whether individual receptor components were required for specific metabolic effects [29]. This approach moves mechanistic inference closer to a controlled perturbation: the more closely two molecules are matched for sequence-dependent properties, exposure, and non-target receptor activity, the more informative the remaining receptor difference becomes.

For triagonists, the ideal experimental framework would extend this strategy to a matched molecular series in which GIPR, GLP-1R, or GCGR activity is systematically attenuated while the remaining receptor pharmacology and pharmacokinetic exposure are preserved. Receptor-selective antagonism or genetic perturbation can provide complementary evidence, particularly in preclinical models. Convergence across matched ligands, receptor perturbation, structural pharmacology, and physiological measurements would provide stronger causal attribution than any single approach alone. These complementary designs are summarized in Figure 5.

### 8.2. Energy Intake, Energy Expenditure, and Metabolic Adaptation

The contribution of GCGR to retatrutide-associated weight reduction is particularly difficult to resolve because body weight reflects the cumulative interaction between energy intake, energy expenditure, nutrient absorption, physical activity, and changes in body composition. GLP-1R and GIPR signaling can influence feeding behavior [13,57,59], whereas experimental GCGR pharmacology provides a mechanistic basis for altered substrate utilization and energy expenditure [17,27,28,29]. Determining their relative contributions therefore requires direct longitudinal measurements rather than inference from body-weight change alone.

A mechanistic human study could evaluate energy balance at complementary stages of treatment. Early measurements, obtained before substantial divergence in body weight, would be particularly informative for detecting pharmacological effects on food intake, substrate oxidation, or energy expenditure that precede major changes in body composition. Later measurements, performed after clinically meaningful weight loss, could determine whether treatment modifies the expected metabolic adaptation to reduced body mass [5,6]. Standardization of recent energy intake, dietary protein, physical activity, and measurement conditions would be necessary because these variables influence both glucagon physiology and energy expenditure [19,20].

Indirect calorimetry can quantify resting energy expenditure and respiratory exchange ratio, whereas accelerometry and free-living energy-expenditure methods provide complementary information about physical activity and total energy requirements. These measurements should be interpreted alongside longitudinal fat mass and fat-free mass because absolute energy expenditure normally decreases during weight loss as metabolically active tissue is reduced [5,6]. A pharmacological attenuation of adaptive thermogenesis could therefore manifest as a smaller-than-expected decline in energy expenditure, rather than an absolute increase above pretreatment values.

The most informative mechanistic endpoint would consequently integrate measured energy intake, absolute and body-composition-adjusted energy expenditure, physical activity, and longitudinal body composition. Such a design could test whether GCGR-containing polyagonism modifies the energetic adaptation to weight loss and quantify the extent to which changes in intake and expenditure contribute to the observed phenotype.

### 8.3. Direct Hepatic Pharmacology and Systemic Weight-Loss Effects

The liver provides a second major setting in which receptor-specific causal attribution remains unresolved. GCGR is highly relevant to hepatic metabolism and regulates glucose production, amino acid catabolism, ureagenesis, fatty acid oxidation, and ketogenesis [19,20,44]. Retatrutide and survodutide produce substantial imaging-based reductions in hepatic steatosis [78,79], making direct hepatic GCGR signaling an attractive mechanistic explanation. However, hepatic substrate delivery also changes rapidly when food intake decreases, insulin sensitivity improves, and adipose-tissue mass is reduced.

Temporal resolution can help distinguish these mechanisms. Changes in hepatic substrate flux occurring before substantial weight loss would provide stronger evidence for an early pharmacological effect than measurements obtained only after large differences in body weight have emerged. Stable-isotope tracer studies could quantify endogenous glucose production, lipolytic substrate delivery, hepatic lipid synthesis, fatty acid oxidation, and amino acid turnover, while serial MRI-PDFF could characterize the subsequent trajectory of hepatic steatosis [19,20,78].

Matched pharmacological comparators would strengthen this approach further. A triagonist and a comparator with closely matched GIPR/GLP-1R activity and exposure but attenuated GCGR activity could test whether GCGR contributes incrementally to early hepatic substrate remodeling. Histological studies would address a different level of disease biology by distinguishing steatosis reduction from MASH resolution and fibrosis improvement [78,80,81].

Importantly, weight loss itself lies on the causal pathway between treatment and several hepatic outcomes. Statistical adjustment for achieved weight loss can therefore answer an association-based question but cannot reproduce a randomized experiment in which GCGR activity differs while other pharmacological components remain constant. Combining temporal phenotyping, tracer physiology, matched receptor pharmacology, imaging, and histology would provide a substantially stronger framework for determining how direct hepatic receptor activity and systemic energy balance jointly contribute to liver outcomes.

### 8.4. Convergent Strategies for Causal Attribution

No single experimental platform can fully resolve receptor-specific contributions to multi-agonist pharmacology. Structural studies define molecular recognition; cellular assays characterize receptor activity and signaling; matched ligands and receptor perturbation test causal dependence; metabolic physiology measures integrated organ-level responses; and randomized clinical trials establish therapeutic efficacy. These approaches address different levels of biological organization and become most informative when their conclusions converge.

For retatrutide, the unresolved question is therefore not whether GIPR, GLP-1R, and GCGR are pharmacologically active—the molecular and functional evidence establishes that they are [31,43]—but how their relative contributions change across tissues, exposure levels, metabolic states, and clinical endpoints. This distinction provides the mechanistic basis for considering receptor balance as an Activity–Exposure–Context problem rather than as a fixed potency ratio.

## 9. The Activity–Exposure–Context Framework for Receptor Balance

The concept of receptor balance is central to multi-receptor drug design, but its biological meaning extends beyond a fixed ratio of in vitro potencies. For a unimolecular polyagonist, the integrated response emerges from at least three interacting dimensions: the intrinsic pharmacology of the molecule at each receptor, the exposure achieved at receptor-accessible sites over time, and the biological context in which those receptors operate. We therefore propose an Activity–Exposure–Context (AEC) framework for interpreting receptor balance across experimental systems and clinical populations.

### 9.1. Limitations of a Single Potency Ratio

Receptor balance is frequently described using relative EC_50_ values across the component receptors of a multiagonist. Such measurements are useful for molecular characterization but remain dependent on assay system, receptor abundance, signal amplification, reference ligand, and experimental duration [31,41,43]. Normalization to the cognate endogenous ligand provides an informative receptor-specific reference—for example, the relative activities of retatrutide at GIPR, GLP-1R, and GCGR shown in Figure 3—but does not place the three receptors on a common physiological scale [31,43].

The in vivo phenotype of a polyagonist therefore cannot be represented by potency alone. A receptor activated with lower potency in vitro may remain biologically important under sustained systemic exposure, whereas high potency at another receptor may be modified by receptor abundance, desensitization, tissue accessibility, or downstream amplification. Receptor balance is consequently better conceptualized as a dynamic relationship between molecular activity, pharmacokinetic exposure, and biological context rather than as a single invariant potency ratio.

### 9.2. Activity: Receptor Recognition, Efficacy, and Regulation

The Activity dimension encompasses the molecular and cellular properties that determine how a ligand engages and activates each receptor. These include binding affinity, functional potency, maximal response, signaling kinetics, pathway preference, receptor internalization, recycling, and sustained signaling [41,43,52,69]. For multi-receptor agonists, these properties should ideally be characterized across all intended targets using comparable experimental platforms and cognate reference ligands.

A single cAMP EC_50_ captures only one dimension of this pharmacology. Two ligands with similar cAMP potency can differ in maximal response, β-arrestin recruitment, receptor trafficking, or duration of signaling [52,69]. Conversely, lower acute potency can coexist with prolonged biological activity when receptor internalization or recycling differs. These observations are particularly relevant to GLP-1R and GCGR pharmacology and provide a mechanistic basis for distinguishing receptor activation from the broader temporal pharmacological phenotype of a ligand.

Biased agonism represents a specialized component of Activity. Formal evidence of signaling bias requires comparison of at least two signaling pathways relative to an appropriate reference agonist under controlled experimental conditions [52,69]. Accordingly, cAMP efficacy, β-arrestin recruitment, and receptor internalization should be reported as distinct properties. This distinction is especially relevant to the clinical GLP-1R/GCGR co-agonists discussed in Section 5, for which pathway-specific signaling and trafficking have been characterized to different degrees.

### 9.3. Exposure: Receptor Activity Across Time and Tissue

The Exposure dimension describes the concentration–time profile of pharmacologically available ligand at receptor-accessible sites. For long-acting peptide agonists, systemic exposure is shaped by dose, absorption, albumin association, distribution, proteolytic stability, clearance, and dosing interval [31,41,101]. Lipidation and related conjugation strategies therefore influence not only apparent half-life but also the temporal conditions under which receptor pharmacology is expressed.

This distinction is particularly important for multiagonists. Two molecules with similar receptor-potency relationships in vitro can generate substantially different in vivo responses if their concentration–time profiles differ. Similarly, the relative contribution of individual receptors may change across the dosing interval if their activation thresholds, receptor reserve, desensitization, or tissue accessibility differ. Receptor balance may therefore be time dependent even when the molecular sequence of the drug remains unchanged.

Activity and exposure are also experimentally interdependent. Chemical modifications introduced to prolong half-life can alter peptide–receptor interactions or the fraction of ligand available for receptor engagement, while increasing dose can modify the duration and magnitude of receptor activation. Pharmacological characterization should therefore integrate concentration–response relationships with pharmacokinetic measurements rather than treating receptor potency and clinical dose as independent descriptors [28,29,41].

### 9.4. Context: Biological Determinants of Phenotypic Expression

The Context dimension encompasses the cellular, tissue, metabolic, and clinical conditions under which receptor activity and exposure are translated into physiology. Relevant variables include receptor abundance, β-cell functional reserve, insulin sensitivity, nutritional state, hepatic disease, body composition, neural circuitry, and the physiological adaptations that accompany progressive weight loss [19,20,44,57,58,59,103,104].

GCGR provides a clear example. Hepatic substrate mobilization produced by GCGR activation may have a different net glycemic consequence when β-cell function and incretin-stimulated insulin secretion are preserved than when insulin secretory capacity is severely impaired [19,20,101,102]. GIPR provides another example: agonism and antagonism can converge on weight reduction through different cellular and neural mechanisms depending on exposure and receptor location [57,58,59,103,104]. Thus, the same molecular receptor activity need not generate an identical organism-level phenotype across metabolic states.

Clinical development of retatrutide provides a translational illustration of this principle. Substantial weight reduction occurs both in populations with and without T2DM, but the magnitude of response differs numerically across phase 3 populations [102,108,109,110,113]. Because these studies were not randomized comparisons of metabolic states, they do not quantify a diabetes-specific modifier of treatment efficacy; nevertheless, they illustrate why disease phenotype should be incorporated into mechanistic interpretation rather than treated as a passive background variable.

The therapeutic objective itself is part of Context. A receptor profile optimized for glycemic control may differ from one optimized for obesity, hepatic disease, or preservation of physical function. Receptor balance should therefore be evaluated relative to a defined therapeutic objective and its associated efficacy, tolerability, and safety constraints rather than against a universal “optimal” GLP-1R:GIPR:GCGR ratio [115].

### 9.5. Integration of Activity, Exposure, and Context

Activity, Exposure, and Context are analytically separable but biologically interdependent. Molecular modifications alter receptor activity and exposure; exposure determines the duration and concentration range over which receptor activity is expressed; and biological context determines how those signals are translated into cellular, organ-level, and clinical responses. The AEC framework therefore conceptualizes receptor balance as a dynamic pharmacological phenotype rather than a fixed molecular constant (Figure 6).

This framework also generates experimentally testable questions. Does a given receptor-activity profile retain the same relative contribution across the dosing interval? Does GCGR activity produce different hepatic or glycemic consequences according to β-cell reserve or liver phenotype? Do baseline enteroinsular responses identify biological contexts in which GIPR-, GLP-1R-, or GCGR-containing pharmacology is expressed differently? Addressing these questions requires integration of receptor pharmacology, pharmacokinetics, metabolic phenotyping, and longitudinal clinical outcomes. Table 4 summarizes the corresponding measurement domains.

## 10. Precision Polyagonism, Response Heterogeneity, and Research Priorities

The clinical development of multi-receptor agonists has demonstrated substantial average improvements in body weight, glycemic control, and metabolic health, yet these population-level effects coexist with considerable interindividual variability [11,12,78,106]. As polyagonist pharmacology becomes increasingly tunable, a major translational challenge will be to determine whether this heterogeneity reflects differences in drug exposure, receptor pharmacology, metabolic state, treatment persistence, or interactions among these factors. The Activity–Exposure–Context framework developed in Section 9 provides a structure for investigating this variability prospectively and for moving from population-level efficacy toward mechanistically informed precision pharmacology.

### 10.1. Response Heterogeneity as a Dynamic Phenotype

Interindividual response to metabolic pharmacotherapy is multidimensional. Changes in body weight, HbA1c, hepatic steatosis, appetite, tolerability, body composition, and physical function need not occur in parallel, and the magnitude of response depends on dose achieved, duration of exposure, treatment persistence, and baseline metabolic phenotype [11,12,78,106,116]. Consequently, categorical labels such as “superresponder” and “nonresponder” may obscure continuous and time-dependent treatment trajectories.

Timing is particularly important when defining response. A post hoc analysis of SURMOUNT-1 demonstrated that some participants with limited early weight reduction during tirzepatide treatment subsequently achieved clinically meaningful weight loss with continued therapy [116]. The analysis included participants who received at least 75% of assigned doses and had weight measurements at baseline and weeks 12, 24, and 72, limiting generalization to patients with interrupted treatment or missing follow-up [116]. Early response therefore does not necessarily define the eventual magnitude of treatment effect, particularly during dose escalation. Longitudinal trajectories provide more information than a single threshold and should be prioritized when investigating biological heterogeneity.

Once differences in achieved dose, pharmacokinetic exposure, adherence, and treatment duration are accounted for, residual variability may reflect biological factors including β-cell functional reserve, insulin sensitivity, central and peripheral receptor responsiveness, adaptive energy expenditure, body composition, hepatic phenotype, and neuroendocrine responses to nutrient ingestion [5,6,31,116]. These variables map naturally onto the Activity–Exposure–Context framework and provide candidate dimensions for prospective metabolic phenotyping.

### 10.2. Mixed-Meal Phenotyping as a Testable Precision-Pharmacology Strategy

A standardized mixed-meal tolerance test (MMTT) provides an experimentally tractable approach for characterizing the enteroinsular component of biological Context before and during polyagonist therapy. Nutrient challenges simultaneously engage glucose-dependent insulin secretion, glucagon regulation, endogenous incretin release, gastrointestinal nutrient handling, and β-cell responsiveness, thereby generating a dynamic physiological phenotype that cannot be captured by fasting hormone concentrations alone [40,117].

The use of mixed-meal challenges in metabolic drug development is already established. Controlled cotadutide studies incorporated mixed-meal glycemia and related metabolic measurements as pharmacodynamic endpoints [87,88], while standardized MMTTs have been evaluated for reproducible assessment of β-cell function [117]. We propose extending this paradigm by testing whether pretreatment and early-treatment enteroinsular responses provide incremental predictive information for subsequent response to multi-receptor agonists.

A candidate protocol would standardize meal energy and macronutrient composition, fasting duration, ingestion time, concomitant medications, and sampling conditions. We propose serial measurement of plasma glucose, C-peptide, insulin, glucagon, and total and intact GLP-1 and GIP. Prior work supports reproducible measurement of selected β-cell-function endpoints [117] and analyte-specific protection against ex vivo peptide degradation [118], not validation of this complete panel for selecting a polyagonist. OXM could be incorporated as an exploratory analyte when an analytically specific assay is available, given the substantial sequence overlap among OXM, glucagon, and glicentin discussed in Section 3 [61,65,66]. A representative sampling scheme could include 0, 15, 30, 60, 90, and 120 min, with extension to 180 min in protocols designed to capture delayed nutrient absorption (Table 5).

Analytical rigor is essential because total and intact incretin concentrations represent different biological quantities influenced by secretion, degradation, and clearance; they are not direct measurements of receptor sensitivity or of secretion and degradation rates [66,118]. Collection procedures should be validated for the intended analyte and include appropriate protection from ex vivo degradation, rapid temperature-controlled processing, standardized storage, and assay-specific assessment of cross-reactivity [65,66,118]. Candidate physiological features could include glucose and hormone area-under-the-curve (AUC) measurements, C-peptide responses interpreted relative to the glucose stimulus, postprandial glucagon suppression, and temporal relationships among incretin secretion and insulin response.

Implementation at scale requires an explicit analytical budget. Paired intact and total GLP-1/GIP measurements multiply the number of assays and sample aliquots, while analyte-appropriate stabilization, rapid processing, frozen transport, batch controls, and cross-platform validation add operational demands [118,119]. Total and intact assays differ in molecular specificity and cannot be substituted without changing the biological question [119]. A feasible staged design could obtain a core glucose/C-peptide panel with standardized clinical and nutritional measurements in the full cohort, while collecting appropriately stabilized samples for paired intact/total incretin and glucagon assays in a prespecified mechanistic subset. Assay expansion and denser sampling should depend on demonstrated reproducibility and incremental predictive value. Subset selection should be specified prospectively to limit selection bias, and costs should be evaluated per participant and per informative endpoint before multicentre implementation.

The central hypothesis is that a reproducible dynamic enteroinsular phenotype may explain a portion of interindividual response heterogeneity beyond routinely measured clinical variables. Demonstrating association with an outcome would represent an initial step; treatment selection would require evidence that the biomarker provides incremental predictive information and identifies differential response between therapeutic alternatives. Prospective studies should therefore incorporate standardized treatment and titration, achieved exposure, adherence, prespecified outcomes, biomarker-by-treatment interaction testing, and external validation.

This hypothesis is directly testable. Enteroinsular phenotyping may improve prediction of weight, glycemic, hepatic, or tolerability responses, or it may add little once drug exposure, disease severity, and early treatment response are considered. Either result would clarify the biological determinants of polyagonist response and define the potential role of dynamic nutrient testing in precision metabolic pharmacology.

### 10.3. Engineering Principles for Next-Generation Polyagonists

The next generation of polyagonists should be designed to test defined physiological hypotheses rather than to maximize receptor number. The causal-attribution framework described in Section 8 provides one strategy: matched molecular series can systematically vary GCGR, GIPR, or GLP-1R activity while preserving the remaining receptor pharmacology and exposure as closely as possible [28,29]. Such experiments could determine whether modification of a specific receptor component alters energy expenditure, hepatic substrate flux, glycemic control, body composition, or tolerability.

Future molecular engineering may also distinguish among receptor selectivity, signaling selectivity, and tissue exposure. These represent related but mechanistically distinct strategies. Tissue-directed delivery modifies where a ligand becomes pharmacologically available; biased agonism modifies the relative signaling pathways engaged after receptor activation; and changes in ligand residence, receptor internalization, recycling, or subcellular signaling can modify the temporal organization of receptor activity [41,52,69]. Each strategy therefore requires molecular, cellular, physiological, and ultimately clinical validation.

The liver illustrates the importance of this distinction. Increasing hepatic exposure to a GCGR agonist could enhance effects on lipid oxidation and amino acid metabolism while simultaneously increasing hepatic glucose production [19,20]. Likewise, reduced β-arrestin recruitment or altered receptor internalization in an experimental system may modify signaling duration without necessarily improving clinical efficacy or tolerability [52,69]. The relevant translational question is therefore whether a defined molecular modification improves a prespecified benefit–risk phenotype under sustained exposure.

Additional receptor combinations should similarly be justified by a defined physiological objective rather than by receptor count alone. The trajectory from OXM to retatrutide demonstrates that conserved Class B1 receptor architecture creates molecular opportunities for polyagonism; medicinal chemistry determines how those opportunities are engineered, and clinical pharmacology determines whether they translate into therapeutic benefit [30,31,41]. The most informative next-generation strategy may therefore be a carefully controlled change in receptor activity, signaling, or exposure rather than the addition of another receptor target.

### 10.4. Quality and Durability of Metabolic Response

As pharmacological weight reduction approaches magnitudes historically associated primarily with metabolic surgery, evaluation of treatment response should extend beyond maximum mean body-weight change. Relevant dimensions include persistence of treatment effect, body composition, physical function, nutritional status, glycemic stability, hepatic disease activity, tolerability, and ultimately cardiovascular, renal, hepatic, and mortality outcomes.

For T2DM, profound improvement in HbA1c during active pharmacotherapy represents therapeutic control rather than drug-free remission. The international consensus defines remission using an HbA1c below 6.5% measured at least three months after cessation of glucose-lowering pharmacotherapy [120]. This distinction becomes increasingly relevant as multi-receptor agonists produce HbA1c concentrations below conventional diagnostic thresholds in substantial proportions of treated participants [102,109].

For metabolic liver disease, reduction or normalization of hepatic fat by MRI-PDFF, histological MASH resolution, fibrosis improvement, and prevention of cirrhosis-related clinical events represent distinct levels of therapeutic evidence [78,80,81]. Their temporal trajectories may differ, emphasizing the importance of sufficiently long follow-up and endpoint-specific interpretation. A relative reduction of ≥80% in MRI-PDFF is therefore not a substitute for complete MASH resolution or stage-specific fibrosis regression. Hepatic lipid clearance may occur rapidly, whereas structural extracellular-matrix remodeling is slower, variable, and potentially incomplete; longer follow-up must test fibrosis and clinical events directly rather than infer them from steatosis alone.

Profound weight loss also increases the importance of determining which tissues are being lost and which functions are preserved. Longitudinal assessment of body composition should therefore be integrated with dietary protein intake, physical activity, muscle strength, and functional performance. This question is particularly relevant to glucagon-containing pharmacology because GCGR signaling participates directly in hepatic amino acid uptake, catabolism, and nitrogen disposal [19,44].

Ultimately, precision polyagonism will require simultaneous optimization of several dimensions: magnitude and durability of metabolic benefit, tolerability, functional tissue preservation, organ-specific disease modification, and long-term clinical outcomes. The next major advance may therefore arise not from adding another receptor, but from determining which receptor activity, at what exposure, and in which biological context produces the most favorable therapeutic phenotype. Figure 7 summarizes the receptor combinations and principal mechanistic similarities and differences underlying these therapeutic strategies. Their unresolved translational constraints are considered in Section 11.

## 11. Challenges and Limitations of Metabolic Polyagonism

### 11.1. Molecular Trade-Offs and Translational Uncertainty

Polyagonist engineering couples several design problems within one molecule. A substitution or conjugation that improves stability or activity at one receptor can change the remaining receptor profile, protein binding, or tissue access. Unimolecular delivery also limits independent titration of the component activities: increasing dose increases exposure to the complete peptide, rather than selectively adjusting GCGR, GIPR, or GLP-1R engagement. Functional EC_50_ ratios and static active-state structures therefore provide important characterization but cannot define tissue occupancy, a universal receptor balance, or the fraction of clinical benefit attributable to each receptor [31,41,42,43].

Species and experimental context further constrain translation. Receptor abundance, neural circuitry, substrate flux, and the energetic response to glucagon differ across systems. Native OXM established biological plausibility but its rapid clearance and early short-duration human studies could not predict the long-term performance of engineered analogues [24,25,61]. Rodent evidence of increased energy expenditure or receptor-dependent liver benefit must therefore be tested using human physiology and appropriate comparators, rather than inferred from the magnitude of clinical weight loss [27,28,29,78,90].

### 11.2. Unmet Endpoints and Limits of Clinical Inference

The development history includes clinically important setbacks and discordant endpoints. GCGR antagonism improved glycemia but increased hepatic fat, aminotransferases, or blood pressure in specific LY2409021 studies, illustrating the consequences of disrupting a pleiotropic endocrine pathway [75,76,77]. Within GCGR-containing agonism, pemvidutide improved MASH resolution at 24 weeks in IMPACT but did not meet the coprimary fibrosis-improvement endpoint [81]. These results identify limits of particular interventions and endpoints; they do not demonstrate inevitable failure of the receptor strategy or establish that longer treatment will reverse fibrosis. Likewise, favorable liver biomarkers with cotadutide did not constitute biopsy-confirmed antifibrotic efficacy [89].

Whole-drug efficacy also leaves important causal questions unanswered. Cross-trial differences cannot establish that a third receptor confers superiority over a dual agonist, and concomitant weight loss complicates attribution of liver effects to direct GCGR signaling. Imaging-defined steatosis reduction, histological disease modification, and prevention of liver events require separate evidence. Sponsor announcements can provide timely information but do not replace complete peer-reviewed methods, uncertainty estimates, and endpoint analyses. These distinctions are particularly important when interpreting extension populations, different estimands, and exploratory cardiovascular analyses [78,81,107,108,109,110,111,112,113].

### 11.3. Long-Term Safety, Function, and Implementation

Gastrointestinal intolerance, dose escalation, and discontinuation can limit the exposure achieved outside trial settings [105,106,108]. Long-term evaluation must also determine whether metabolic benefit is sustained alongside adequate nutrition and physical function, especially in older adults or people with low functional reserve. Lean-mass measurements alone cannot establish muscle preservation, and GCGR-mediated amino acid turnover provides a rationale for testing nitrogen handling without assuming that increased ureagenesis proves harmful muscle catabolism [19,44,114]. Risk-factor improvements and large weight reductions remain insufficient to establish cardiovascular, renal, or liver-event benefit for an individual polyagonist. Access, treatment costs, adherence, and the feasibility of repeated physiological testing will also influence implementation and generalizability.

### 11.4. Evidentiary Limits of the AEC Framework

AEC is an organizing framework, not a validated predictive model, causal decomposition, or prescribing algorithm. Its domains interact, some variables are difficult to measure in accessible human tissues, and several proposed biomarkers may add little beyond routine clinical features, achieved exposure, or early response. MMTT-derived hormone concentrations cannot directly identify receptor sensitivity or separate secretion from clearance. A useful treatment-selection tool would require reproducibility, prespecified treatment-by-biomarker interactions, calibration, external validation, and evidence that the added complexity improves decisions [117,118,119].

Finally, this review uses targeted narrative retrieval and selective synthesis rather than exhaustive study identification, formal risk-of-bias assessment, or quantitative meta-analysis. The supplementary historical-term search improves hepatic retrieval but cannot eliminate selection or publication bias. These limitations support treating the mechanistic and precision-pharmacology proposals as hypotheses for prospective testing, while separating them from the clinical effects already demonstrated in randomized trials.

## 12. Conclusions

OXM provides an endogenous pharmacological precedent for simultaneous GLP-1R and GCGR activation and illustrates how conserved structural relationships within the Class B1 GPCR family can support cross-receptor peptide recognition [21,34,35,42]. Medicinal chemistry transformed this biological precedent into tunable GLP-1R/GCGR co-agonism by modifying receptor activity, molecular stability, and pharmacokinetic exposure [28,29]. The resulting clinical programs established that GCGR agonism, historically regarded primarily as a diabetogenic liability, can be incorporated into metabolically effective therapies when integrated with incretin receptor activation.

Retatrutide represents the most advanced clinical expression of this strategy by combining GIPR, GLP-1R, and GCGR agonism within a single long-acting peptide [31,43]. Phase 2 and phase 3 studies demonstrate that this pharmacology can produce profound reductions in body weight together with substantial improvements in glycemic control and metabolic phenotypes across obesity and T2DM [78,102,105,106,107,108,109,110,113]. At the same time, the diversity of responses across populations, the distinct development trajectories of GLP-1R/GCGR co-agonists, and the GIPR agonism–antagonism paradox demonstrate that receptor combination alone is insufficient to explain the clinical phenotype.

We therefore propose that receptor balance is better understood as an Activity–Exposure–Context relationship: molecular receptor activity defines what a ligand can engage, pharmacokinetic exposure determines when and for how long that activity is expressed, and biological context determines how receptor signaling is translated into physiology. Within this framework, standardized mixed-meal phenotyping provides a testable strategy for investigating whether dynamic enteroinsular responses contribute predictive information to interindividual treatment heterogeneity. The framework remains conceptual; its utility depends on prospective validation, practical measurement strategies, and demonstration of value beyond established clinical predictors.

The next phase of polyagonist development should therefore move beyond increasing receptor number toward mechanistically defined receptor activity, controlled exposure, and prospective identification of the biological contexts in which those properties produce the greatest therapeutic benefit. The trajectory from OXM to retatrutide suggests that the future of metabolic polyagonism may depend less on discovering additional targets than on learning how to engineer—and eventually personalize—the interactions among receptors already embedded within conserved endocrine networks.

## Figures and Tables

**Figure 1:**
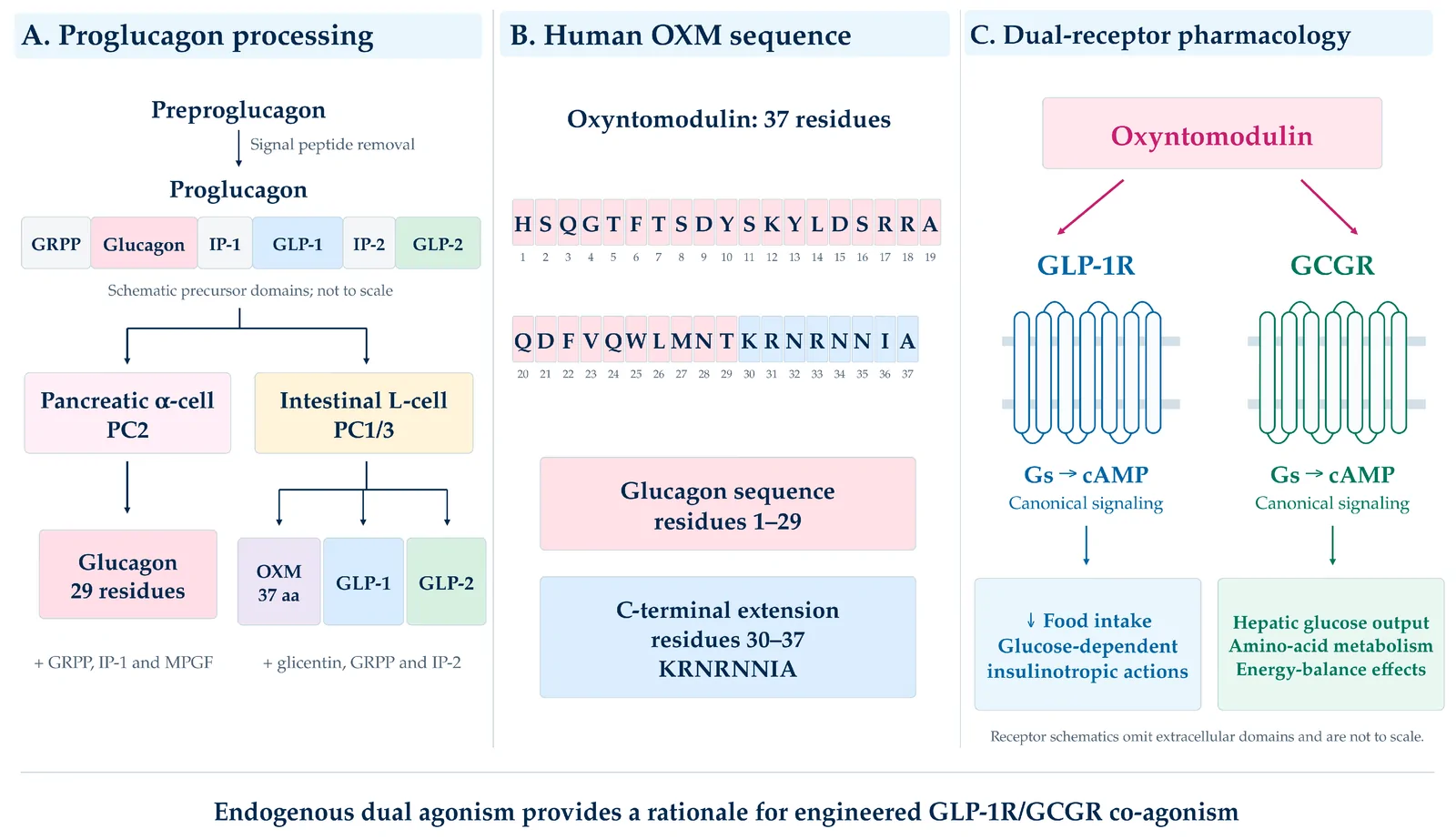
Biosynthesis, molecular identity, and dual-receptor pharmacology of oxyntomodulin. (**A**) Tissue-specific proglucagon processing. (**B**) Human oxyntomodulin (OXM) sequence. (**C**) Representative receptor-associated metabolic actions. Arrows in (**A**) indicate precursor processing; magenta arrows in (**C**) indicate receptor activation, and blue/green arrows connect signaling to representative effects. The downward-arrow symbol beside food intake denotes a decrease. Schematic receptor domains and qualitative arrows do not indicate potency, receptor occupancy, or human effect sizes. GRPP, glicentin-related pancreatic polypeptide; IP-1/2, intervening peptides 1/2; MPGF, major proglucagon fragment; PC, prohormone convertase [21,22,23,27,39,42,53,54,55,60,61].

**Figure 2:**
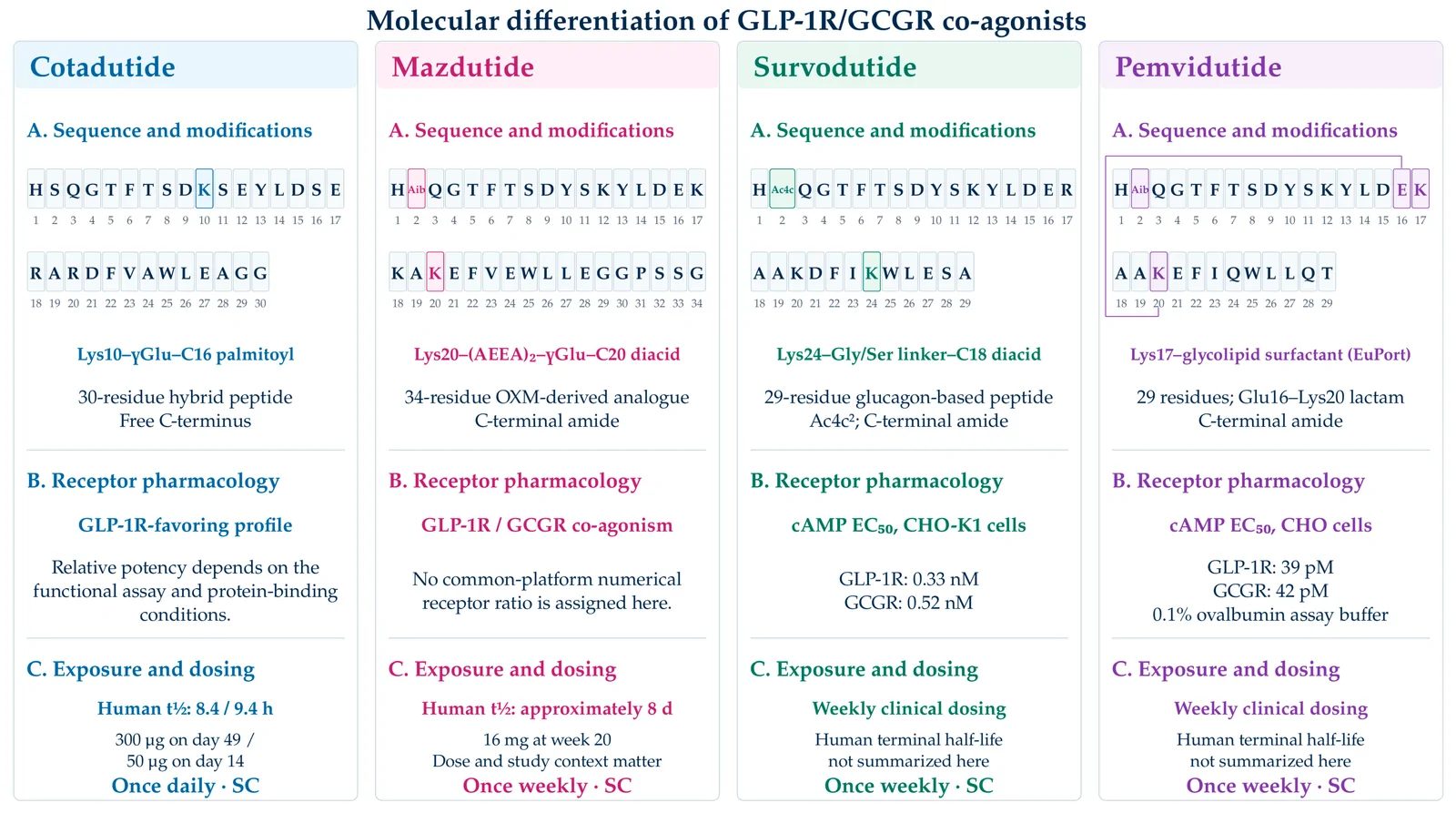
Molecular differentiation of GLP-1R/GCGR co-agonists. Peptide sequences, conjugation sites, functional assay contexts, and exposure strategies of cotadutide, mazdutide, survodutide, and pemvidutide. Colored compound panels distinguish the four co-agonists. Colored residues denote specified noncanonical or chemically modified residues, outlined residue boxes mark modified or conjugated sites, and the purple connecting line denotes the Glu16–Lys20 lactam. Linker and lactam connectivity are schematic rather than atom-resolved chemical structures. Assay-specific potencies do not establish a cross-molecule ranking. Half-lives refer to the dose and study context indicated in each panel. Aib, α-aminoisobutyric acid; AEEA, 2-[2-(2-aminoethoxy)ethoxy]acetic acid; Ac4c, 1-aminocyclobutane-1-carboxylic acid; cAMP, cyclic adenosine monophosphate; CHO, Chinese hamster ovary; EC_50_, half-maximal effective concentration; GCGR, glucagon receptor; GLP-1R, glucagon-like peptide-1 receptor; OXM, oxyntomodulin; SC, subcutaneous; t½, half-life; γGlu, γ-glutamyl [84,85,86,87,88,91,92,93,94,95,97,98].

**Figure 3:**
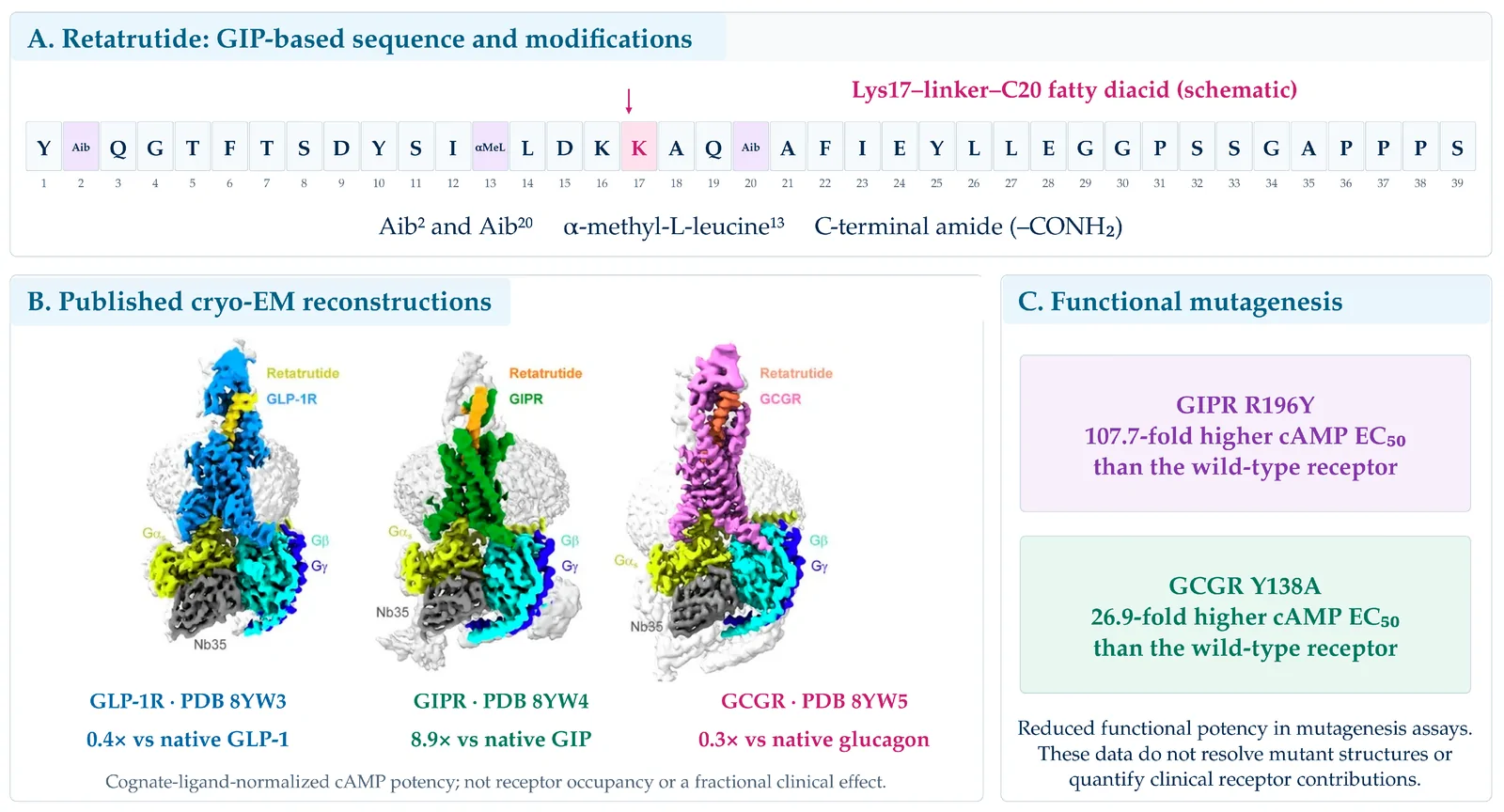
Retatrutide: GIP-based molecular architecture and triple-receptor recognition. (**A**) Sequence and modifications of the 39-residue GIP-based peptide, including Aib^2^ and Aib^20^, α-methyl-L-leucine^13^, Lys^17^-linked C20 fatty-diacid conjugation, and C-terminal amidation; linker connectivity is schematic. The magenta arrow marks the Lys17 conjugation site, shaded residue boxes indicate noncanonical or modified sites, and the colored boxes in (**C**) distinguish the GIPR and GCGR assays. Retatrutide was engineered from a GIP peptide backbone [31]. OXM is a conceptual precedent for receptor co-agonism, not its structural precursor. (**B**) Published Gs-coupled cryo-EM reconstructions of retatrutide bound to GLP-1R, GIPR, and GCGR (PDB 8YW3, 8YW4, and 8YW5, respectively), with receptor-specific cAMP potency ratios normalized to the corresponding native ligands [31,43]. These in vitro EC_50_-based ratios do not translate linearly into in vivo fractional receptor occupancy or clinical efficacy and do not represent percentages of receptor engagement. (**C**) Functional mutagenesis increased cAMP EC_50_ by 107.7-fold for GIPR R196Y and 26.9-fold for GCGR Y138A relative to the corresponding wild-type receptors [43]. The data do not resolve mutant structures or quantify clinical receptor contributions. Panel (**B**) reproduced, cropped and resized, from Li et al. [43], Cell Discovery 2024, 10, 77, original Figure 1b, under the Creative Commons Attribution 4.0 International (CC BY 4.0) license (https://doi.org/10.1038/s41421-024-00700-0; https://creativecommons.org/licenses/by/4.0/; accessed on 28 October 2026). The original component colors and labels are retained; surrounding panels and annotations are explanatory summaries. Aib, α-aminoisobutyric acid; cAMP, cyclic adenosine monophosphate; cryo-EM, cryo-electron microscopy; EC_50_, half-maximal effective concentration; GCGR, glucagon receptor; GIP, glucose-dependent insulinotropic polypeptide; GIPR, GIP receptor; GLP-1R, glucagon-like peptide-1 receptor; OXM, oxyntomodulin.

**Figure 4:**
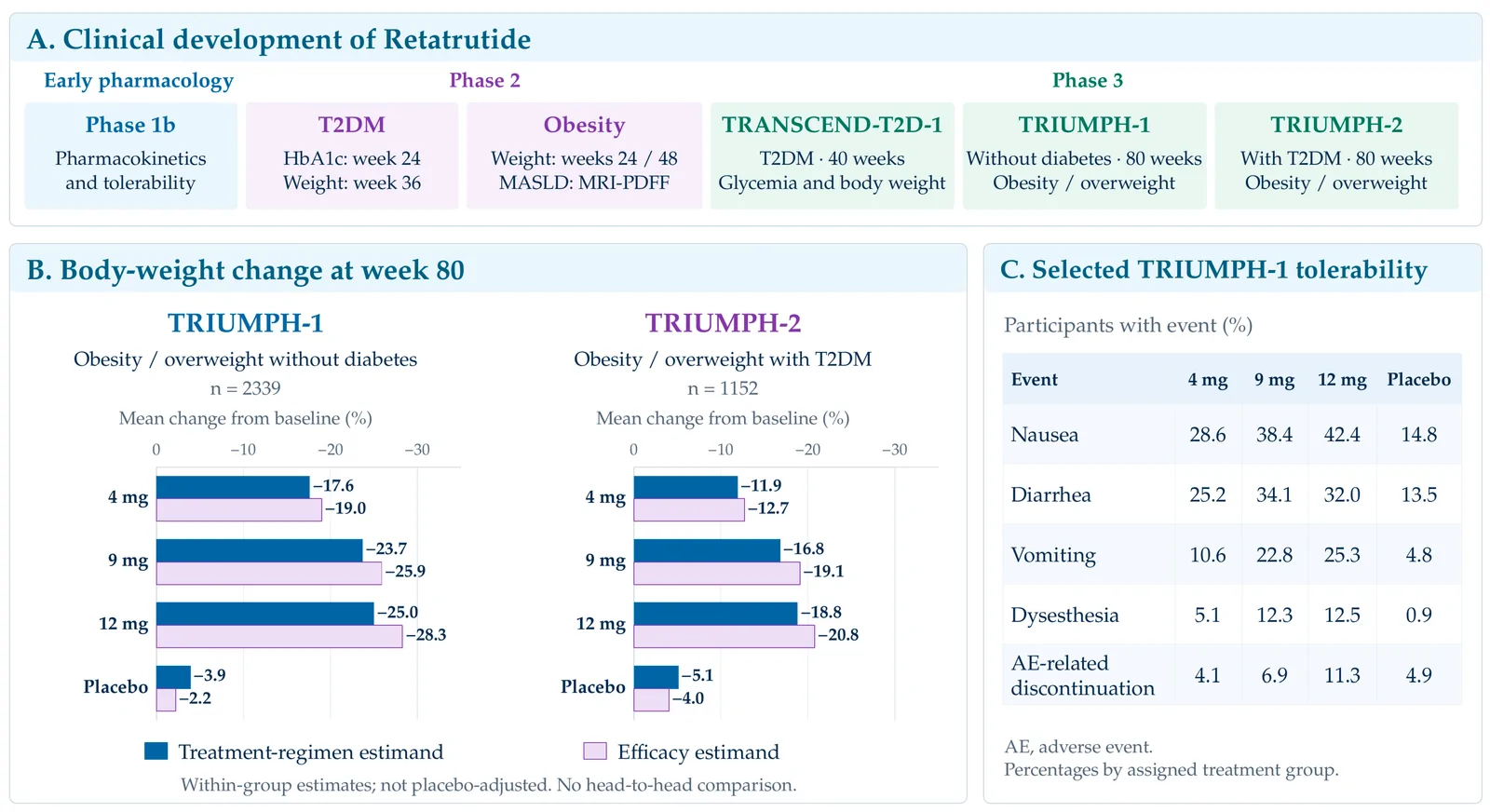
Retatrutide: clinical development, efficacy, and tolerability. (**A**) Selected clinical milestones. (**B**) Mean within-group body-weight changes at week 80 in TRIUMPH-1 and TRIUMPH-2 by treatment-regimen and efficacy estimand. Values are not placebo-adjusted, and the display does not constitute a head-to-head comparison between trial populations. The efficacy estimand addresses a hypothetical scenario of remaining on treatment without prohibited weight-loss medication, rather than a completer-only analysis. (**C**) Selected TRIUMPH-1 adverse events and adverse-event-related discontinuation, expressed as percentages within assigned treatment groups. Point estimates are shown without uncertainty intervals. AE, adverse event; HbA1c, glycated hemoglobin; T2DM, type 2 diabetes mellitus; MASLD, metabolic dysfunction-associated steatotic liver disease; MRI-PDFF, magnetic resonance imaging proton density fat fraction [78,101,102,105,106,107,108,109,111,112].

**Figure 5:**
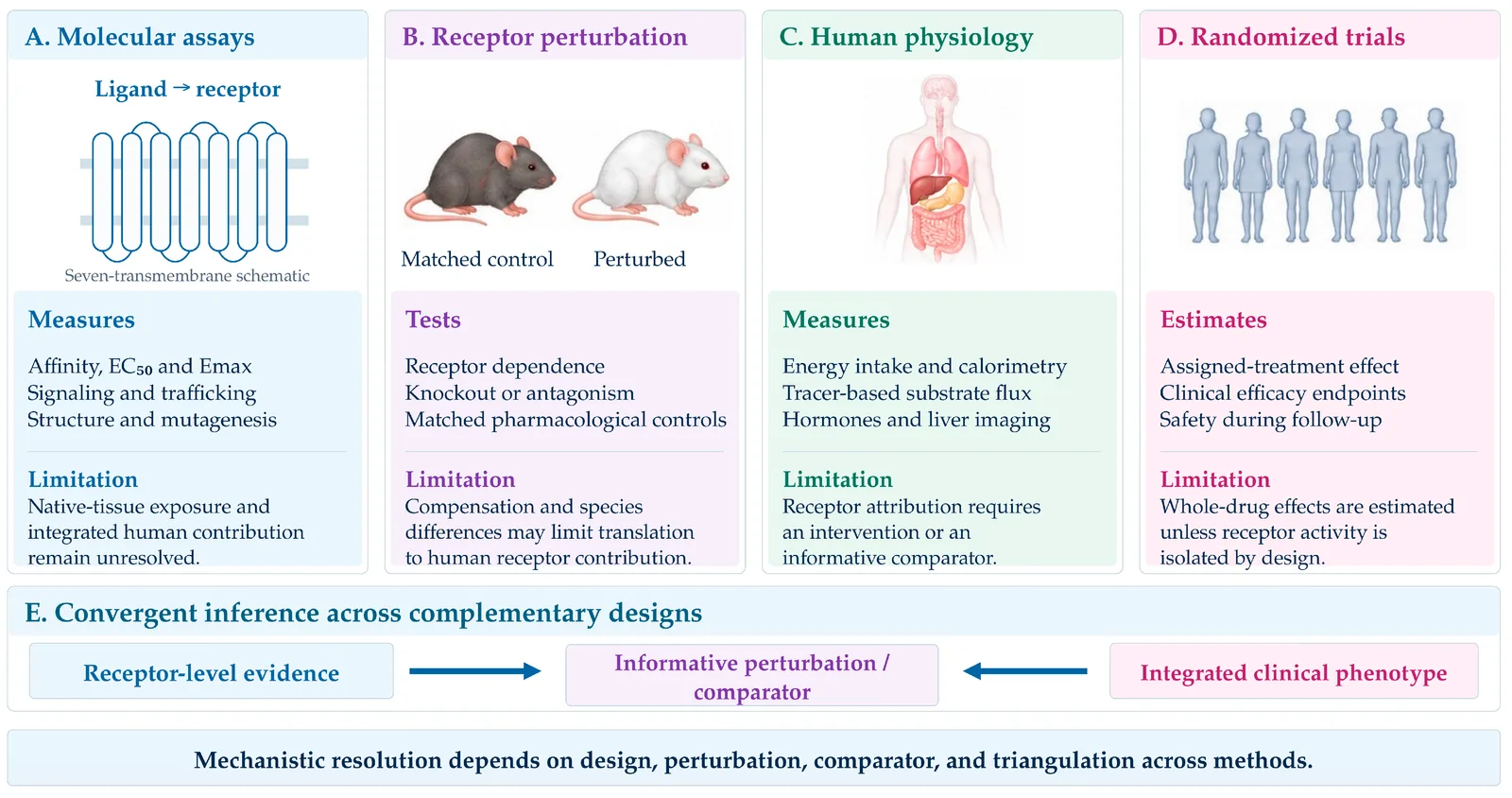
Complementary designs for receptor-specific causal attribution. (**A**) Molecular assays, (**B**) receptor perturbation, (**C**) human physiology, and (**D**) randomized trials provide complementary information; panel colors distinguish the study designs. Opposing arrows in (**E**) indicate convergence through an informative perturbation or comparator, not the direction or magnitude of an effect. Drug efficacy can be established without identifying the fraction of benefit mediated by an individual receptor. (**E**) Receptor-specific attribution requires an informative intervention or comparator and convergence across methods. Coat color alone does not establish a valid matched experimental control or receptor perturbation. EC_50_, half-maximal effective concentration; Emax, maximum measured response [23,27,28,29,43,57,59,69,78,106].

**Figure 6:**
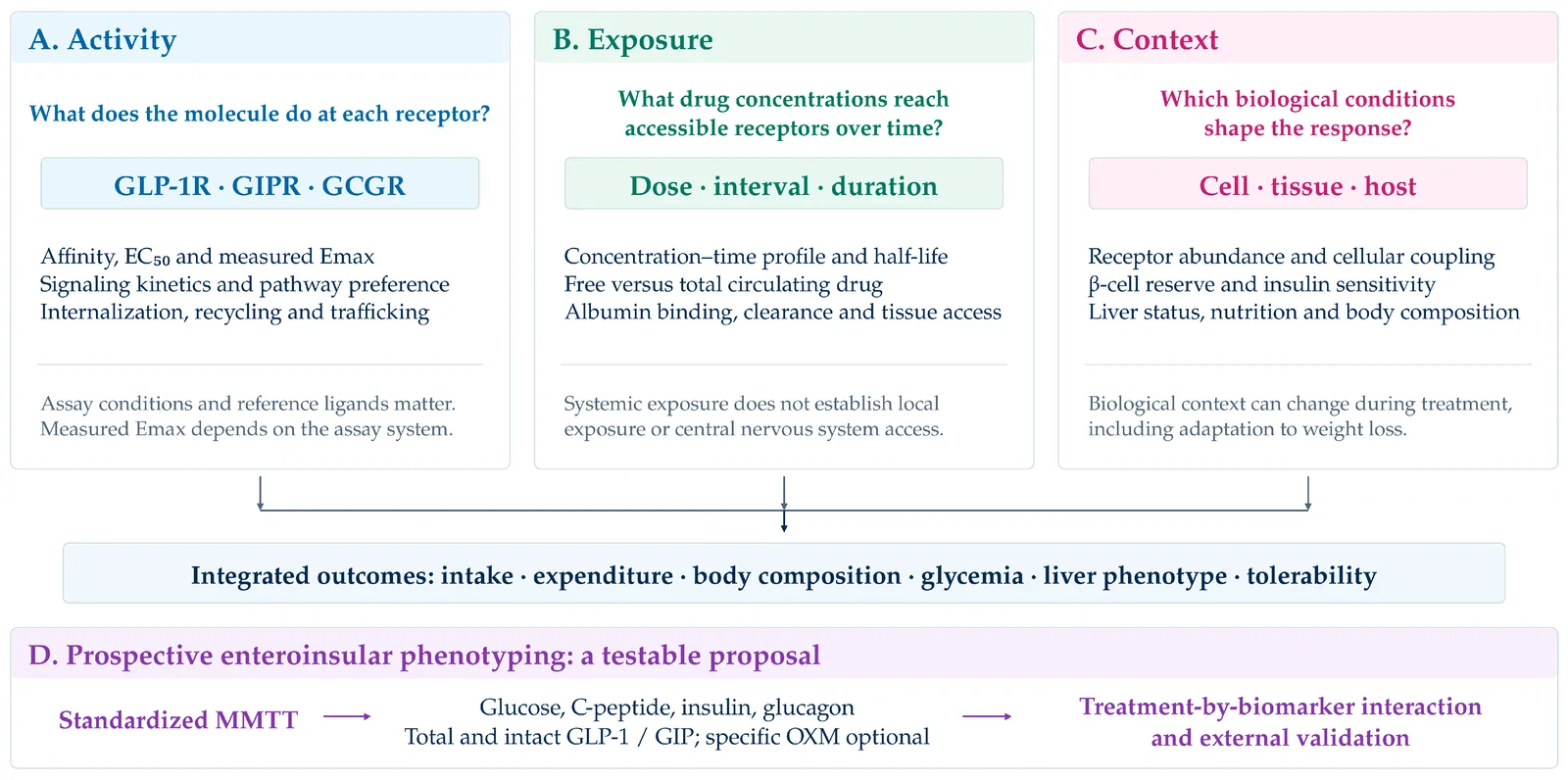
Proposed Activity–Exposure–Context framework. (**A**) Activity, (**B**) exposure, and (**C**) biological context jointly influence integrated outcomes, as indicated by the converging arrows. Purple arrows in (**D**) show the proposed phenotyping and validation sequence. This is a conceptual framework, not a validated score, multiplicative equation, or treatment-selection algorithm. (**D**) Prospective enteroinsular phenotyping is a testable proposal requiring treatment-by-biomarker interaction testing and external validation. Hormone concentrations do not directly measure receptor sensitivity. Prognostic association is not treatment-specific prediction. AEC, Activity–Exposure–Context; EC_50_, half-maximal effective concentration; Emax, maximum measured response; GCGR, glucagon receptor; GIP, glucose-dependent insulinotropic polypeptide; GIPR, GIP receptor; GLP-1, glucagon-like peptide-1; GLP-1R, GLP-1 receptor; MMTT, mixed-meal tolerance test; OXM, oxyntomodulin [19,31,41,57,58,59,69,88,103,104,116,117,118].

**Figure 7:**
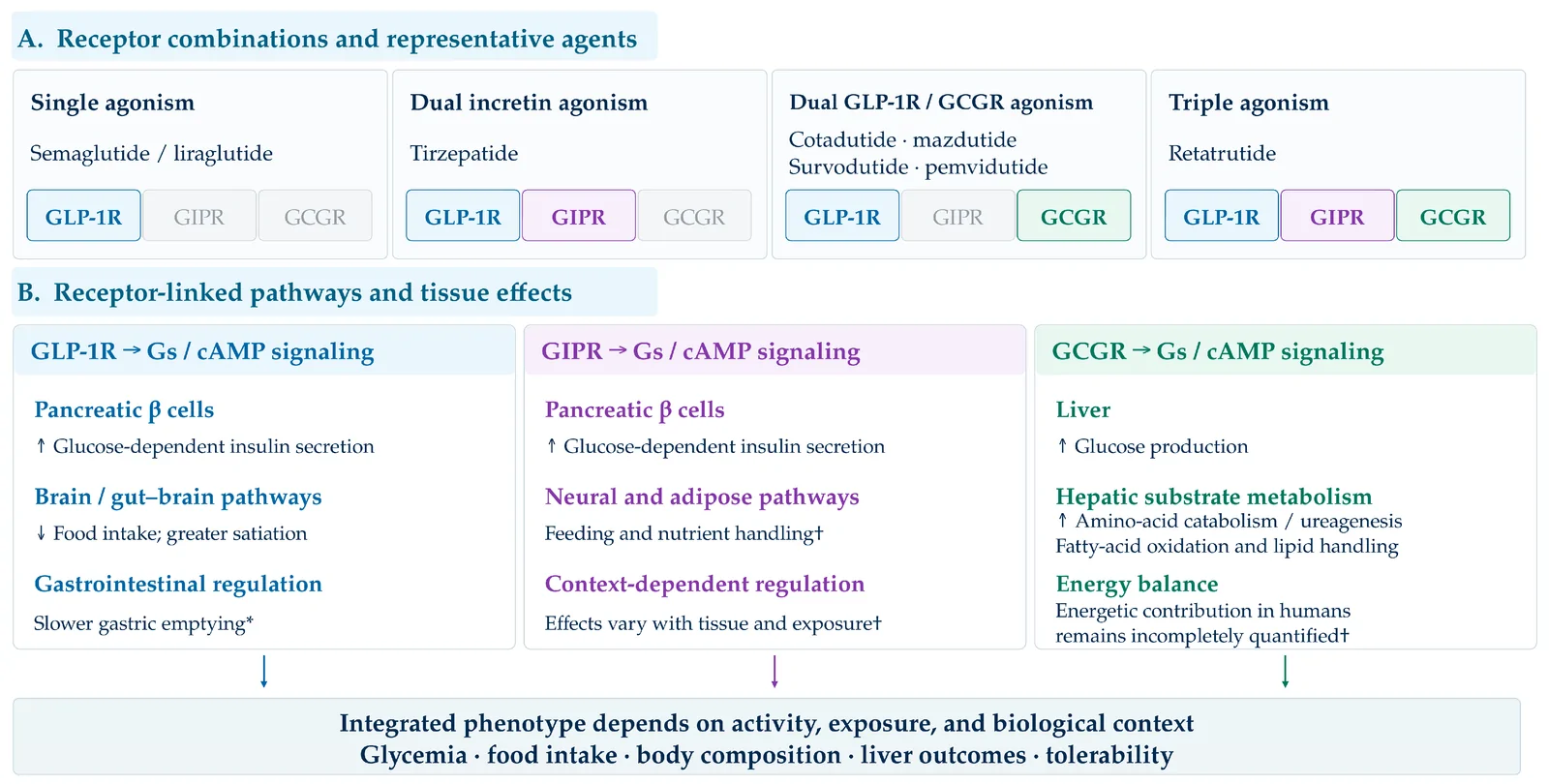
Single, dual, and triple receptor agonism. (**A**) Receptor combinations and representative agents. Blue, purple and green indicate GLP-1R, GIPR and GCGR, respectively; gray receptors are not intended agonist targets. Upward and downward symbols beside physiological outcomes denote increases and decreases; connecting arrows indicate signaling or conceptual convergence. (**B**) Receptor-linked pathways and tissue effects. The classification concerns receptor targets, not peptide scaffold origin. Retatrutide is GIP-based and activates GLP-1R, GIPR, and GCGR. * Gastric effects depend on the molecule, dose, and treatment duration. † Mechanistic evidence includes preclinical studies; quantitative human contributions are not established by this diagram. Receptor count does not establish clinical superiority. Arrows and receptor colors do not denote potency, occupancy, or effect size. cAMP, cyclic adenosine monophosphate; GCGR, glucagon receptor; GIP, glucose-dependent insulinotropic polypeptide; GIPR, GIP receptor; GLP-1R, glucagon-like peptide-1 receptor [8,11,12,19,20,28,29,30,31,57,58,59,69,87,88].

**Table 1:** Early human metabolic studies of native oxyntomodulin.

Study	Design and Population	Principal Finding	Pharmacological Contribution
Cohen et al., 2003 [26]	Acute controlled OXM administration in healthy adults; subsequent ad libitum meal assessment	OXM significantly reduced energy intake during the subsequent meal	Established acute anorectic activity of pharmacological OXM in humans
Wynne et al., 2005 [24]	Repeated subcutaneous native OXM administration for 4 weeks in adults with overweight or obesity	Mean body-weight change was −2.3 kg with OXM vs. −0.5 kg with placebo	Provided early proof of concept that sustained OXM exposure can reduce body weight in humans
Wynne et al., 2006 [25]	Randomized crossover study assessing food intake and components of energy expenditure	OXM reduced energy intake and increased activity-related (~26%) and total (~9%) energy expenditure, without a significant increase in resting energy expenditure	Suggested that the human metabolic actions of OXM may extend beyond appetite suppression
Shankar et al., 2018 [67]	Native OXM administration in adults with obesity, with and without T2DM	OXM produced glucoregulatory effects under the experimental conditions studied	Extended human OXM pharmacology from energy balance to glucose regulation

OXM, oxyntomodulin; T2DM, type 2 diabetes mellitus. Results are reported according to the individual study designs and should not be interpreted as direct efficacy comparisons across studies.

**Table 2:** Representative clinical evidence for GLP-1R/GCGR co-agonists; not a cross-trial efficacy ranking.

Dual Agonist	Main Therapeutic Focus	Trial/Phase	Population and Treatment	Principal Reported Outcomes
Cotadutide (MEDI0382) AstraZeneca/MedImmune, UK/USA	T2DM, overweight/obesity; metabolic and hepatic physiology	NCT02548585, phase 2a [88]	51 randomized adults with T2DM and BMI 27–40 kg/m^2^; once-daily SC cotadutide up to 200 μg; 41 days.	At day 41, post-MMTT glucose AUC decreased 32.78% vs. 10.16% with placebo; body weight decreased 3.84 vs. 1.70 kg, respectively [88]. GI adverse events were more frequent with cotadutide.
Mazdutide (IBI362/LY3305677) Innovent Biologics, China; program originated through collaboration with Eli Lilly, USA	Obesity/overweight and T2DM	GLORY-1, phase 3 [91]	Chinese adults with overweight/obesity; once-weekly SC 4 or 6 mg	At week 48, mean weight change was −11.00% (4 mg), −14.01% (6 mg), and +0.30% placebo; ≥15% weight loss occurred in 35.7%, 49.5%, and 2.0%, respectively [91].
Mazdutide (IBI362/LY3305677) Innovent Biologics, China	Obesity, including participants with and without T2DM	GLORY-2, phase 3 [92]	461 treated Chinese adults with obesity; 16.1% had T2DM; once-weekly SC 9 mg vs. placebo for 60 weeks	Mean weight change at week 60 was −16.65% vs. −1.50% placebo; ≥5% weight loss occurred in 84.3% vs. 33.1%. GI adverse events were predominant [92].
Survodutide (BI 456906) Boehringer Ingelheim, Germany/Zealand Pharma, Denmark	Obesity	SYNCHRONIZE-1, phase 3 [95]	725 adults with obesity without T2DM; once-weekly SC 3.6 or 6.0 mg; 76 weeks	Treatment-regimen estimand: mean weight change −12.2% (3.6 mg), −13.0% (6 mg), vs. −5.4% placebo. ≥5% loss: 72.6%, 71.9%, and 46.3%, respectively [95].
Survodutide (BI 456906) Boehringer Ingelheim, Germany/Zealand Pharma, Denmark	Overweight/obesity with T2DM	SYNCHRONIZE-2, phase 3; NCT06066528 [96]	752 adults with T2DM and BMI ≥ 27 kg/m^2^; once-weekly SC 3.6 or 6.0 mg or placebo; 76 weeks.	Treatment-regimen estimand: mean weight change −8.2% (3.6 mg), −9.8% (6 mg), vs. −3.9% placebo. GI adverse events: 72.8%, 77.7%, and 38.6%, respectively [96].
Survodutide (BI 456906) Boehringer Ingelheim, Germany/Zealand Pharma, Denmark	MASLD/MASH and obesity	SYNCHRONIZE-MASLD, phase 3 [79]	218 randomized adults with obesity and at-risk MASLD (216 treated); once-weekly SC 6 mg or placebo; 48 weeks.	Efficacy estimand: ≥30% relative liver-fat reduction in 84.2% vs. 24.3%; mean weight change −12.2% vs. −1.0%. Both coprimary endpoints were met [79].
Survodutide (BI 456906) Boehringer Ingelheim, Germany/Zealand Pharma, Denmark	Biopsy-confirmed MASH	Phase 2 MASH trial [80]	Adults with biopsy-defined MASH and fibrosis	Demonstrated histological improvement of MASH without worsening of fibrosis, supporting progression of the hepatic program [80].
Pemvidutide (ALT-801) Altimmune, USA	MASH and obesity	IMPACT, phase 2b [81]	212 participants with biopsy-confirmed MASH and F2/F3 fibrosis; once-weekly SC 1.2 or 1.8 mg; 24-week primary analysis	MASH resolution without worsening fibrosis: 58% (1.2 mg), 52% (1.8 mg), vs. 20% placebo. Fibrosis improvement without worsening MASH: 33%, 36%, vs. 28%, respectively; the fibrosis endpoint was not significant [81].

**Table 3:** Clinical development and representative outcomes of retatrutide.

Trial/Study	Phase	Population	Treatment/Duration	Principal Clinical Outcome	Representative Result
T2DM dose-ranging trial (NCT04867785) [105]	2	281 randomized adults with T2DM (275 in the efficacy analysis); placebo- and dulaglutide-controlled.	Retatrutide up to 12 mg once weekly; glycemic assessment at 24 weeks and weight assessment at 36 weeks	Glycemic control and body weight	At 12 mg, HbA1c decreased by 2.02 percentage points at week 24 and body weight by 16.94% at week 36
Obesity dose-ranging trial (NCT04881760) [106]	2	338 adults with obesity or overweight, without T2DM	Retatrutide up to 12 mg once weekly; 48 weeks	Body weight	At 12 mg, mean body-weight change was −24.2% vs. −2.1% with placebo
MASLD substudy of NCT04881760 [78]	2	98 participants with baseline liver fat ≥10%	Retatrutide up to 12 mg once weekly; primary MRI assessment at 24 weeks	Hepatic steatosis by MRI-PDFF	At 12 mg, mean relative liver-fat change was −82.4% vs. +0.3% with placebo
TRANSCEND-T2D-1 [102]	3	537 adults with relatively early T2DM inadequately controlled with diet and exercise	Retatrutide 4, 9, or 12 mg once weekly vs. placebo; 40 weeks	HbA1c, fasting glucose and body weight	At 12 mg, HbA1c change was −1.94 percentage points vs. −0.81 with placebo; body-weight change was −15.3% vs. −2.6%, respectively, at week 40 (treatment-regimen estimand).
TRIUMPH-1 [108]	3	2339 adults with obesity, or overweight plus ≥1 weight-related complication, without diabetes	4, 9 or 12 mg once weekly; 80 weeks	Body weight	Treatment-regimen estimand: −17.6%, −23.7%, −25.0% vs. −3.9% placebo. Efficacy estimand: −19.0%, −25.9%, −28.3% vs. −2.2% placebo
TRIUMPH-1 extension (sponsor report) [107]	3 extension	Participants from TRIUMPH-1 with baseline BMI ≥ 35 kg/m^2^ entering the prespecified extension	Continued treatment through 104 weeks	Long-term body-weight reduction	Mean body-weight reductions approached 30% with the highest-dose strategy during continued treatment
TRIUMPH-2 [109]	3	1152 adults with obesity or overweight and T2DM	4, 9 or 12 mg once weekly; 80 weeks	Body weight and HbA1c	Treatment-regimen estimand: −11.9%, −16.8%, −18.8% vs. −5.1% placebo. Efficacy estimand: −12.7%, −19.1%, −20.8% vs. −4.0% placebo. HbA1c (reported primary analysis): −1.38, −1.50, −1.45 percentage points vs. −0.45. Efficacy-estimand body-weight values are also reported in the detailed sponsor communication [112].
TRIUMPH-3 (sponsor report) [113]	3	Adults with severe obesity and established cardiovascular disease, with or without T2DM	9 or 12 mg once weekly; 80 weeks	Body weight and cardiometabolic risk factors	Efficacy-estimand body-weight reductions reached −21.6% and −22.6% with 9 and 12 mg, respectively, vs. −3.2% with placebo; multiple cardiometabolic risk markers also improved
TRIUMPH-4 (sponsor report) [110]	3	Adults with obesity or overweight and knee osteoarthritis, without diabetes	9 or 12 mg once weekly; 68 weeks	Body weight, knee pain and physical function	Efficacy-estimand body-weight reductions were −26.4% and −28.7% with 9 and 12 mg vs. −2.1% placebo; WOMAC pain and physical function also improved

**Table 4:** Experimental dimensions for characterizing Activity–Exposure–Context in multi-receptor pharmacology.

AEC Dimension	Domain	Core Measurements	Mechanistic Question Addressed
Activity	Receptor pharmacology	Affinity, EC_50_, Emax, cognate reference ligand, receptor expression	How strongly and effectively does the molecule activate each receptor?
Activity	Signaling and trafficking	cAMP kinetics, β-arrestin recruitment, internalization, recycling, sustained signaling	Does receptor activation differ in pathway, magnitude, or duration?
Exposure	Pharmacokinetics	Dose–concentration relationship, half-life, free/total exposure, dosing interval	What receptor-active concentrations are sustained over time?
Context	Energy balance	Intake, expenditure, activity, fat mass, fat-free mass	How is receptor pharmacology translated into whole-body energy balance?
Context	Hepatic metabolism	MRI-PDFF, substrate flux, insulin action, histology where appropriate	How do direct and systemic mechanisms contribute to hepatic outcomes?
Context	Nitrogen and functional tissue	Protein intake, amino acids, nitrogen handling, lean mass, strength/function	How does profound weight loss interact with amino-acid metabolism and functional tissue?
Integrated clinical phenotype	Trial estimand	Population, dose/titration, visit, intercurrent events, discontinuation, confidence intervals	What treatment effect is being estimated and in whom?
Integrated clinical phenotype	Durability and outcomes	Persistence, withdrawal, cardiovascular, renal and hepatic outcomes	Does metabolic efficacy translate into durable clinical benefit?

The AEC framework is proposed in this review as a conceptual structure for integrating molecular pharmacology, pharmacokinetic exposure, biological state, and clinical phenotype. It is not a validated scoring system or formal risk-of-bias instrument.

**Table 5:** Proposed prospective mixed-meal phenotyping strategy: measurements, interpretations and validation requirements.

Domain	Proposed Measurement	Derived Information	Validation Objective
Standardized nutrient challenge	Prespecified energy and macronutrient composition; fixed fasting and ingestion conditions; representative sampling at 0, 15, 30, 60, 90, and 120 min, with an optional extension to 180 min	Dynamic metabolic response to a reproducible nutrient stimulus	Establish within-person reproducibility and sensitivity to metabolic phenotype
Glycemic response	Serial plasma glucose	Excursion, peak, time-to-peak, AUC	Determine incremental association with subsequent glycemic and weight response
β-cell response	C-peptide and insulin interpreted relative to glucose	Stimulus-dependent insulin secretory response	Evaluate whether β-cell functional reserve modifies treatment response
α-cell response	Serial glucagon	Basal concentration and postprandial suppression/recovery	Test whether α-cell/hepatic-axis phenotype modifies response to GCGR-containing therapy
Incretin response	Total and intact GLP-1 and GIP	Concentration–time profiles reflecting secretion, degradation, and clearance; not direct receptor sensitivity.	Test association and treatment-specific predictive value
OXM—exploratory	Analytically specific OXM measurement	Endogenous OXM response to nutrient ingestion	Establish analytical feasibility before evaluating predictive utility
Preanalytical control	Validated collection matrix; protease/DPP-4 protection where appropriate; rapid temperature-controlled processing; standardized storage	Reliable preservation of intended analytes	Minimize degradation and assay-related phenotypic misclassification
Clinical integration	Baseline phenotype, achieved dose/exposure, adherence, and longitudinal outcomes	Multivariable treatment-response phenotype	Evaluate calibration, discrimination, incremental predictive value, and external validity

MMTT, mixed-meal tolerance test; AUC, area under the concentration–time curve. The proposed sampling schedule and biomarker panel constitute a prospective research strategy requiring validation and are not established treatment-selection criteria.

## Data Availability

No new data were created or analyzed in this study. Data sharing is not applicable to this article; all sources used for the synthesis are cited in the manuscript.

## Abbreviations

AEC	Activity–Exposure–Context
AP	area postrema
AUC	area under the curve
cAMP	cyclic adenosine monophosphate
DPP-4	dipeptidyl peptidase-4
EC_50_	half-maximal effective concentration
ECD	extracellular domain
ECL	extracellular loop
Emax	maximal measured response
FGF21	fibroblast growth factor 21
GCGR	glucagon receptor
GIPR	glucose-dependent insulinotropic polypeptide receptor
GLP-1R	glucagon-like peptide-1 receptor
GPCR	G protein-coupled receptor
GRK2	G protein-coupled receptor kinase 2
HbA1c	glycated hemoglobin
MASLD	metabolic dysfunction-associated steatotic liver disease
MASH	metabolic dysfunction-associated steatohepatitis
MMTT	mixed-meal tolerance test
MRI-PDFF	magnetic resonance imaging proton density fat fraction
OXM	oxyntomodulin
PC1/3	prohormone convertase 1/3
PC2	prohormone convertase 2
PK	pharmacokinetics
T2DM	type 2 diabetes mellitus
TMD	transmembrane domain
